# Supplementary material for: Development and Assessment of Tailored Illustrations to Enhance Community Understandings of Genetics Topics
Source: Am J Biol Anthropol. 2026 Jul 20;190(3):e70314. doi: 10.1002/ajpa.70314 (PMC13385646; doi:10.1002/ajpa.70314)
Supplement: Supplementary file 1 — Data S1: ajpa70314‐sup‐0001‐Supinfo1.docx. [file AJPA-190-e70314-s004.docx]

**Part 1: Demographic information**

1. Date
2. Interviewer
3. Local name
4. Name on IC card
5. Sex
6. Can I take a picture?
7. Do you know how old you are?
   1. If yes, age
   2. If no, estimated age
8. Village
9. How many years of schooling have you had?
10. Highest level of schooling *(none, primary, secondary, college)*

**Part 2: Prior knowledge**

1. Would you like to know more about blood?
2. Do you think there is information about health in your blood?
   1. If yes, what type of information do you think is in your blood?

**Part 3: Thoughts after viewing the illustrations:**

1. What image was your favorite?
2. Why was that image your favorite?
3. Were there images you did not understand?
   1. If yes, which ones?
4. If you could learn more about an image, which would it be?
5. What else would you like to know about your blood? *(health & disease/relatedness/specifics about DNA/similarity and differences to other populations/other)*
6. What is one thing you learned after seeing the illustrations?
7. Do you think you could explain at least one of these illustrations to a friend?
8. I think I know more about DNA now than I did before seeing the illustrations
9. The presentation and illustrations helped me understand why a scientist would want to study blood
10. The images helped me understand why researchers want to study Orang Asli
11. The illustrations were hard to understand
12. I would look at the illustrations again
13. I would tell a friend to look at the illustrations
14. I would like to learn more about what I learned today
