# Supplementary material for: Development and Assessment of Tailored Illustrations to Enhance Community Understandings of Genetics Topics
Source: Am J Biol Anthropol. 2026 Jul 20;190(3):e70314. doi: 10.1002/ajpa.70314 (PMC13385646; doi:10.1002/ajpa.70314)

**What is in my blood?**


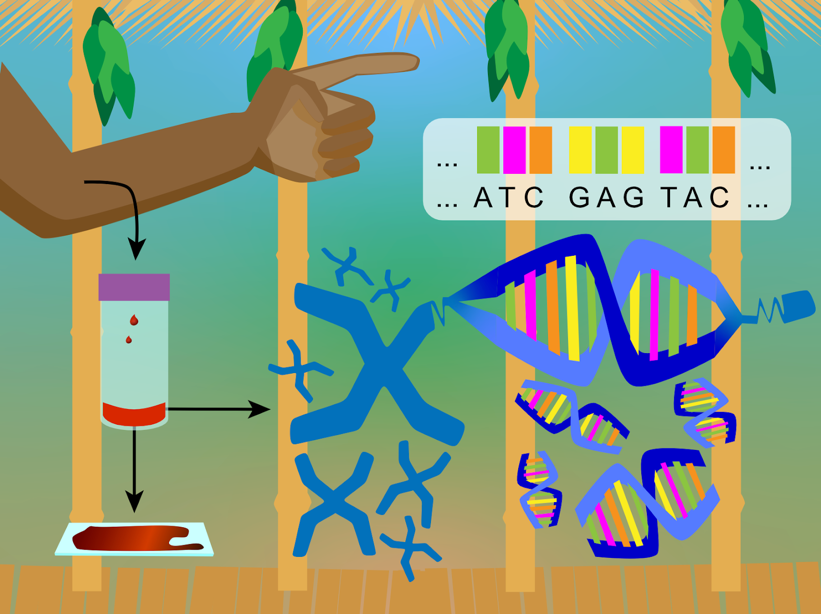


- In the blood there is a special substance called DNA
- DNA can influence your health

**You get your DNA from your parents**


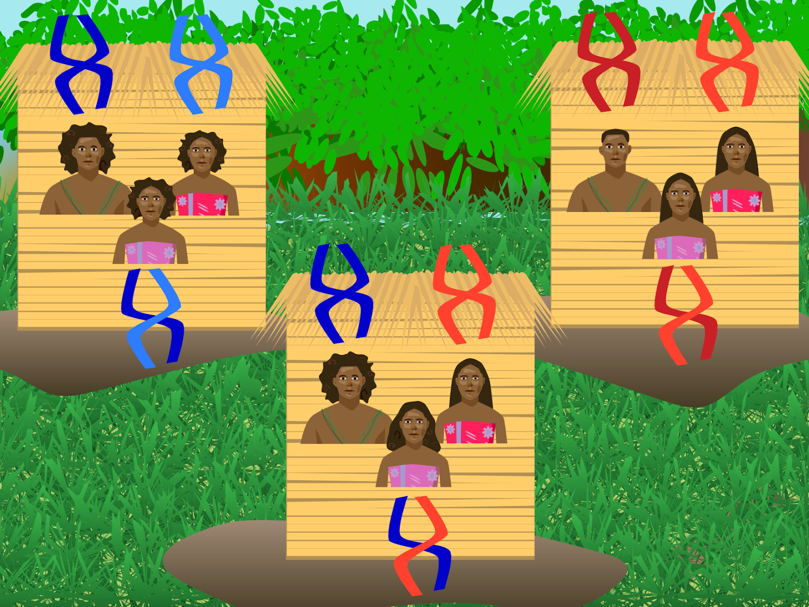


- Everyone gets their DNA from their mother and father
- They get their DNA from their parents (your grandparents)
- Everyone’s DNA is different, which is why we all look different

**All plants and animals also have DNA**


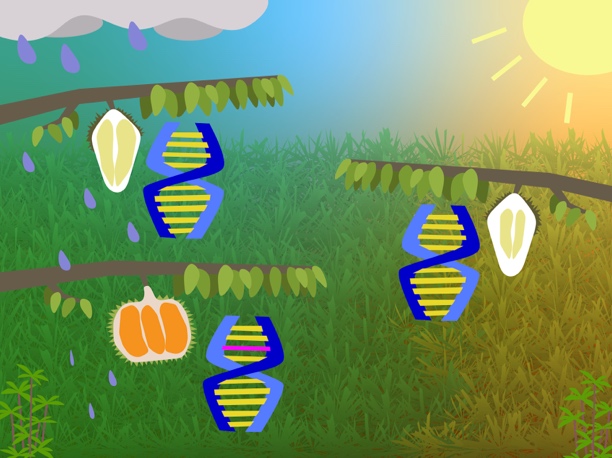


- Differences in DNA contribute to different varieties of durian

**DNA can also affect your health and how you feel**


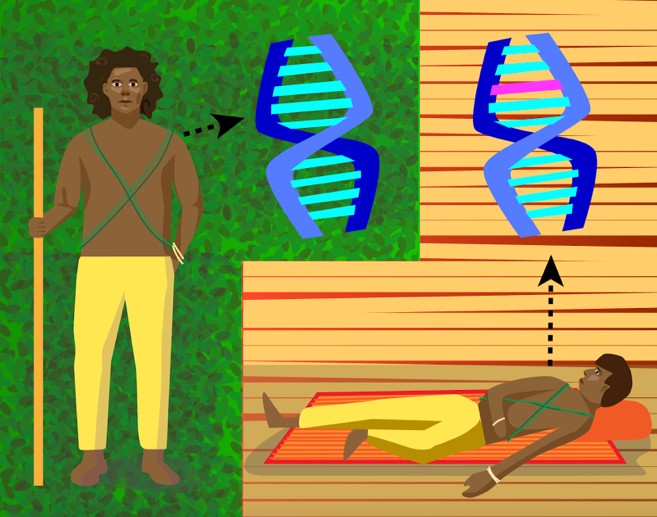


- Sometimes the changes in DNA, called “mutations” can cause disease

**What else can you learn in blood?**


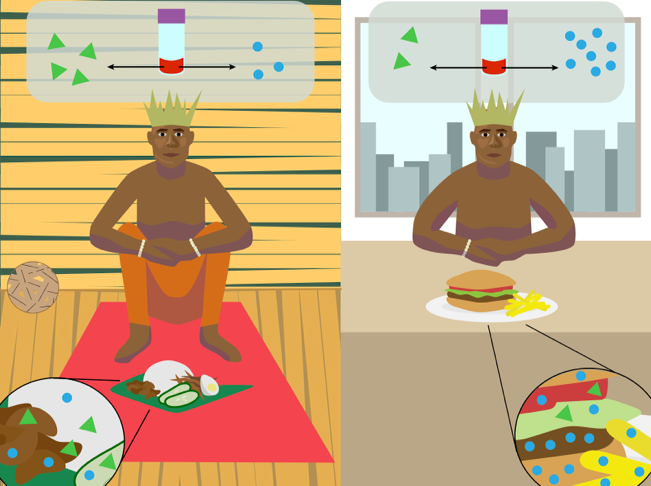


- There are sugars and fats in your blood that come from the food you eat

**Lifestyle affects health**


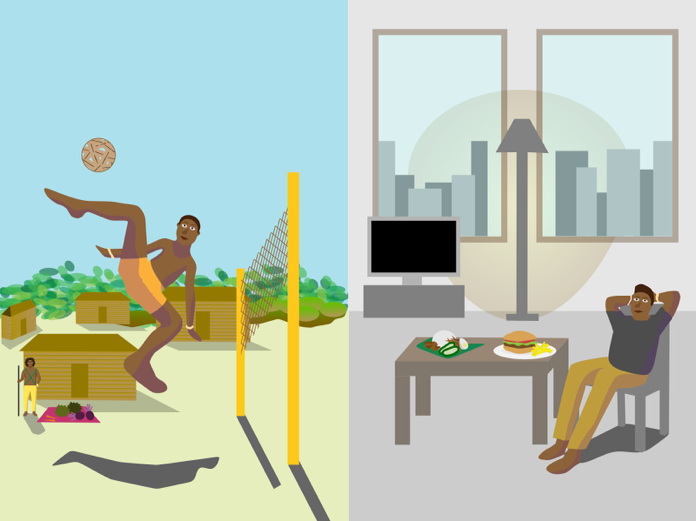


- A more active lifestyle often leads to better health outcomes

**Why are scientists interested in my DNA?**


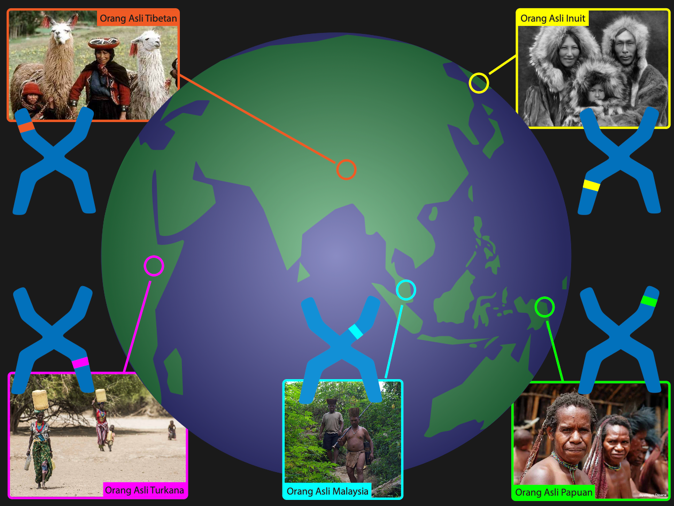


- Some DNA changes contribute to certain populations thriving in their environment
- We can also understand how changes in DNA may cause health problems

**Only scientists from the OA HeLP project will have access to your DNA**


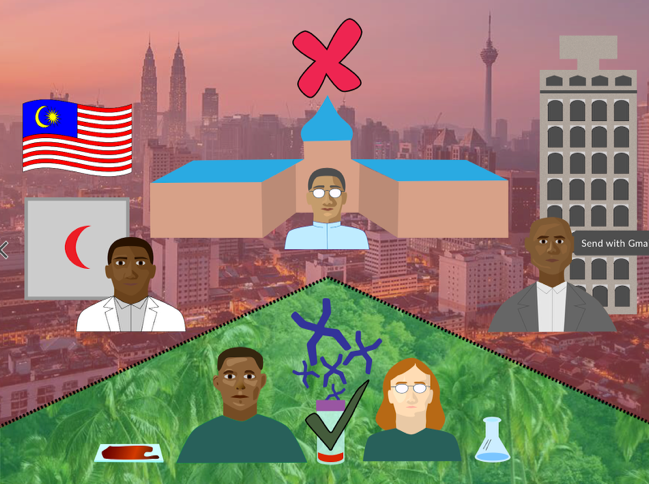

Supplement: Supplementary file 2 — Data S2: ajpa70314‐sup‐0002‐Supinfo2.docx. [file AJPA-190-e70314-s003.docx]
