# Supplementary material for: Development and Assessment of Tailored Illustrations to Enhance Community Understandings of Genetics Topics
Source: Am J Biol Anthropol. 2026 Jul 20;190(3):e70314. doi: 10.1002/ajpa.70314 (PMC13385646; doi:10.1002/ajpa.70314)

**Apakah yang ada dalam**

**darah saya?**


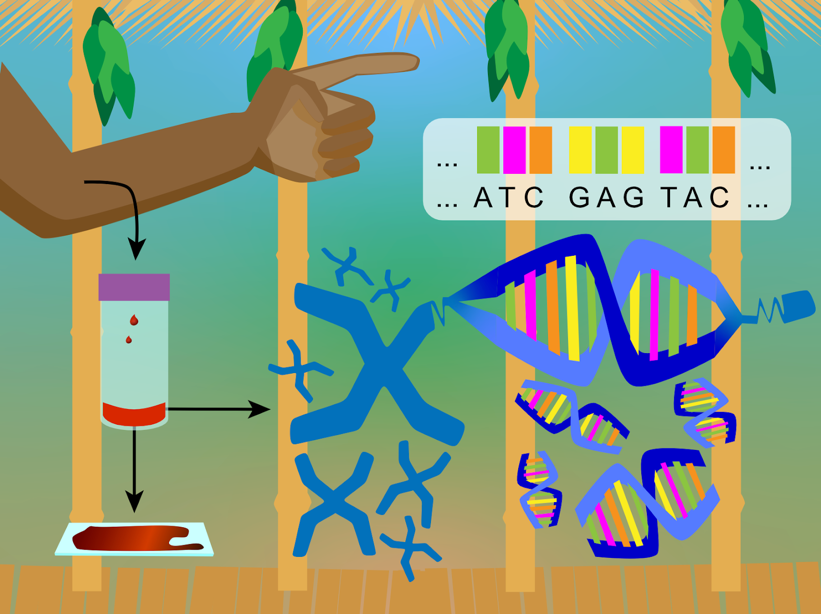


- Di dalam darah terdapat bahan khas yang dipanggil DNA
- DNA mempunyai pengaruh besar terhadap kesihatan anda

**DNA diwarisi daripada ibu bapa**


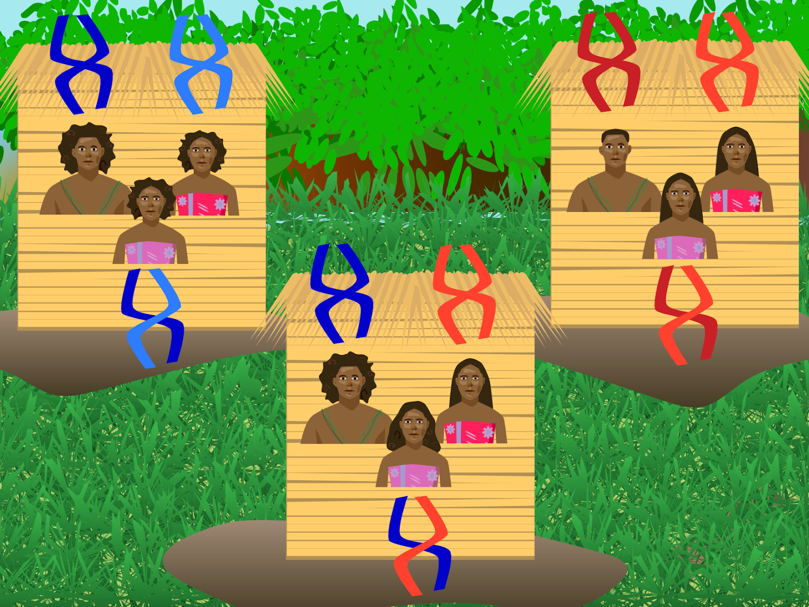


- Kami mendapat DNA kami daripada ibu dan bapa kami,
- Mereka mendapat DNA mereka daripada datuk dan nenek anda
- DNA setiap orang adalah berbeza, itulah sebabnya kita semua tidak kelihatan sama

**Semua tumbuhan dan haiwan lain juga mempunyai DNA**


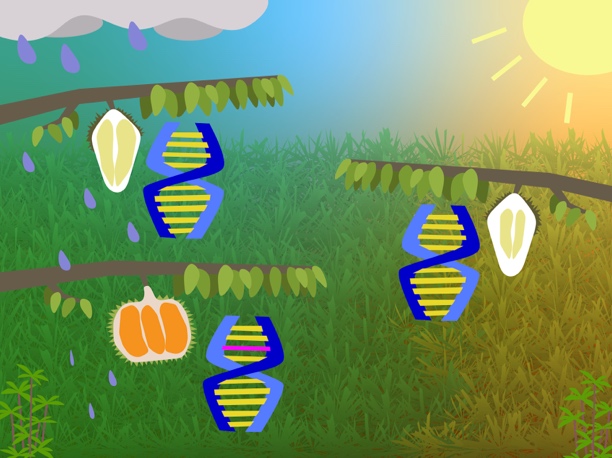


- Perbezaan dalam DNA mewujudkan pelbagai jenis durian

**DNA juga boleh menjejaskan kesihatan anda dan persaan anda**


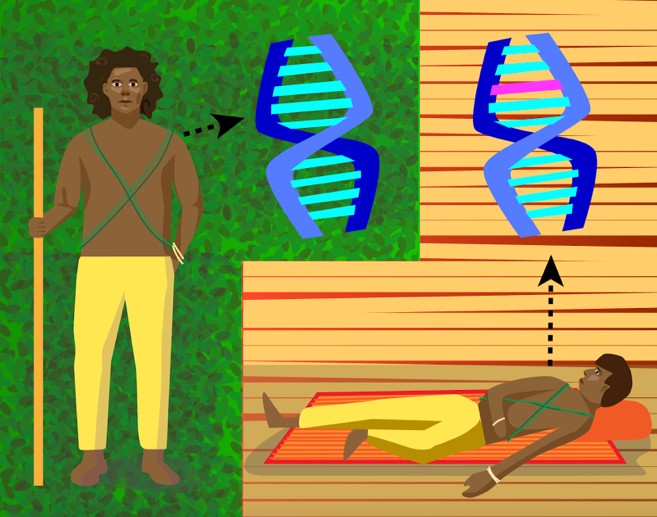


- Kadang-kadang perubahan yang kita lihat dalam DNA, dipanggil "mutasi", boleh menyebabkan penyakit

**Apa lagi yang anda boleh belajar dalam darah?**


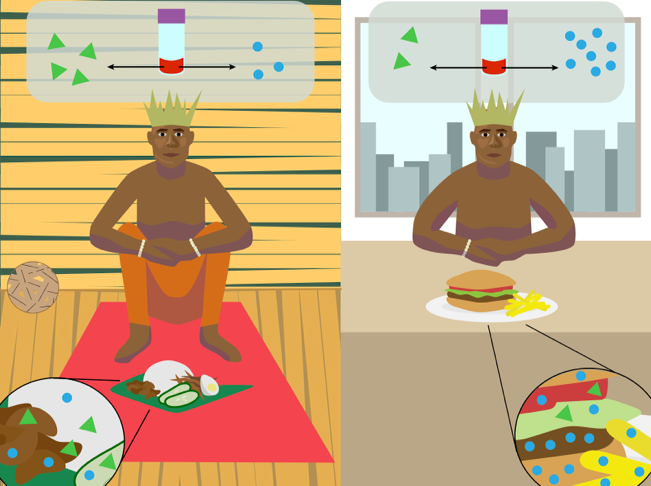


- Terdapat gula dan lemak dalam darah anda yang berasal dari makanan yang anda makan

**Gaya hidup memberi kesan kepada kesihatan**
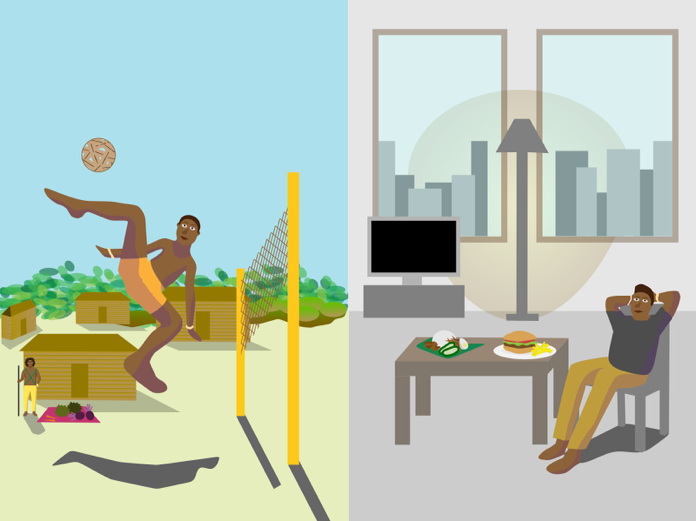


- Gaya hidup yang lebih aktif membawa kepada kesihatan yang lebih baik

**Mengapa saintis berminat dengan DNA asli?**


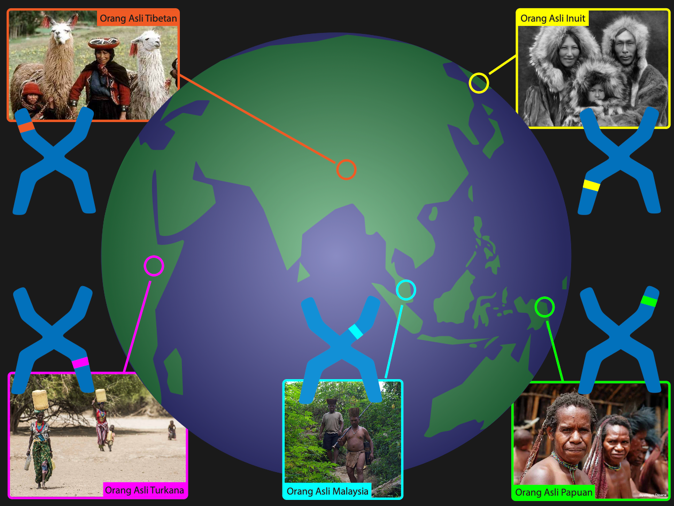


- DNA tertentu boleh membantu kumpulan orang asli hidup dalam persekitaran mereka
- Kita juga boleh memahami bagaimana perubahan dalam DNA menyebabkan masalah kesihatan

**Hanya saintis daripada projek OA HeLP mempunyai akses kepada DNA anda**


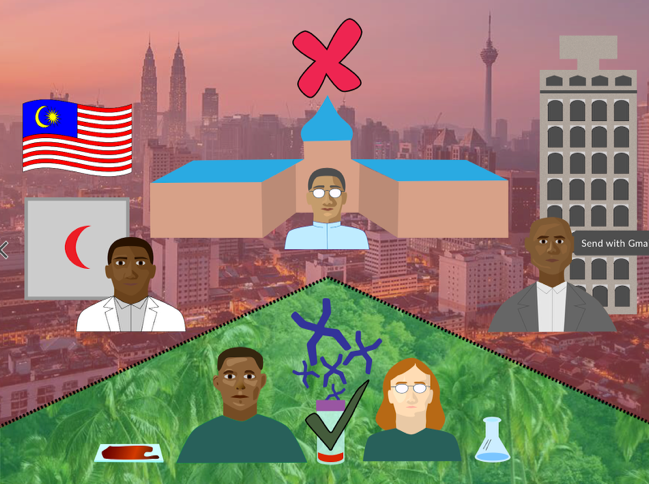

Supplement: Supplementary file 3 — Data S3: ajpa70314‐sup‐0003‐Supinfo3.docx. [file AJPA-190-e70314-s002.docx]
