# Supplementary material for: Development and Assessment of Tailored Illustrations to Enhance Community Understandings of Genetics Topics
Source: Am J Biol Anthropol. 2026 Jul 20;190(3):e70314. doi: 10.1002/ajpa.70314 (PMC13385646; doi:10.1002/ajpa.70314)
Supplement: Supplementary file 4 — Data S4: ajpa70314‐sup‐0004‐Supinfo4.docx. [file AJPA-190-e70314-s001.docx]

**Kichwa: Uundaji na tathmini ya michoro mahususi ili kuboresha uelewaji wa jamii kuhusu mada za jenetiki**

**Jarida Linalolengwa:** Jarida la Marekani la Anthropolojia ya Kibiolojia

**Waandishi:** Audrey M. Arner1, Tobias C. McCabe1, Amanda Seyler2, Siti Nurani Zamri3, Tan Bee Ting A/P Tan Boon Huat3, Kar Lye Tam3, Patriciah Kinyua4, Echwa John4, Sospeter Ngoci Njeru4,5, Yvonne A.L. Lim3, Michael Gurven6, Colin Nicholas7, Vivek V. Venkataraman2, Julien Ayroles8, Thomas S. Kraft9, Ian J. Wallace10, Amanda J. Lea1

**Mawasiliano:** amanda.j.lea@vanderbilt.edu

**Maelezo:**

**Muhtasari**

Malengo

Mawasiliano ya dhati kuhusu dhana za jenetiki ni muhimu kwa utafiti shirikishi wa jenetiki ya anthropolojia. Hata hivyo, mawasiliano yanaweza kuwa changamoto kwa sababu mawazo mengi ni dhahania na yanaweza kuwa mageni hasa kwa jamii zilizofikiwa na kiwango kidogo cha elimu rasmi. Hakika, hakuna mifano iliyokubalika na wengi kwa ajili ya kuwasilisha taarifa hizo, wala huelewa uwazi wa mambo ya kijamii yanayoweza kuathiri ushiriki wa washiriki. Hapa, tulifanya utafiti wa kimaelezo na kiasi, unaoendeshwa na jamii ili kuelewa jinsi michoro inavyoweza kuwa na manufaa kusaidia upashanaji wa dhana na makundi mawili ya kiasili—Watu wa Orang Asli wa Peninsula ya Malaysia na Waturkana wa Kenya.

Mbinu

Tulitumia mbinu ya awamu mbili kuunda na kutathmini jinsi michoro inavyoweza kuimarisha mawasiliano kuhusu dhana za jenetiki. Kwanza, tuliunda picha zinazoelezea majibu ya maswali yanayoulizwa mara kwa mara kuhusu jenetiki, huku tukiziboresha michoro hiyo kulingana na maoni ya washiriki. Pili, tulifanya mahojiano 92 ili kutathmini ufanisi wa michoro iliyokamilika. Hatimaye, tulichambua data ya mahojiano kwa kutumia uchambuzi wa kimaudhui, uundaji wa mifano ya vigezo vingi, na uchambuzi wa mawasiliano mbalimbali ili kutambua mifumo ya uelewaji na maoni ya washiriki, ikijumuisha umri, jinsia, ushirikiano wa kibiashara, na elimu.

Matokeo

Washiriki waliripoti nia ya juu katika utafiti wa jenetiki (92%) na mitazamo chanya kwa upana kuhusu michoro hiyo. Picha zinazofahamika na zenye misingi ya ndani zilipendekezwa na kuhusishwa na uwazi zaidi uliohisiwa, ilhali michoro ya kiufundi zaidi iliripotiwa mara kwa mara kuwa inachanganya. Uchambuzi wa kiasi ulionyesha uthabiti mkubwa wa ndani katika vipimo vya ushiriki na uelewaji, kukiwa na mabadiliko kidogo kulingana na kiwango cha ushirikiano wa kibiashara, elimu, na jinsia.

Majadiliano

Matokeo yetu yanathibitisha kuwa uonyeshaji wa picha mahususi kwa jamii, uliotengenezwa kwa pamoja kupitia maoni ya mara kwa mara, unaweza kusaidia kwa ufanisi ushiriki katika utafiti wa jenetiki ndani ya jamii za washiriki.

Utangulizi

Kuunganisha jenetiki na genomiki katika utafiti wa kibiolojia na kitabibu kumeendeleza uelewaji wetu wa mageuzi ya binadamu, magonjwa, na tofauti za kifenotiki. Hata hivyo, maarifa hayo hayajaenea kwa usawa katika makundi ya watu na yamejikita zaidi kwa watu wenye asili ya Ulaya wanaoishi katika nchi zenye mapato ya juu [1–4]. Umuhimu wa sampuli mbalimbali unatambulika kote kama jambo muhimu kwa uelewaji thabiti wa michakato ya mageuzi na muundo wa kijenetiki wa sifa na magonjwa tata [5,6], na ni muhimu katika kushughulikia tofauti za kiafya na kuongeza ufikiaji wa faida zinazotokana na matokeo ya baadaye [7]. Uelewaji huu umesababisha mipango ya hivi karibuni inayopanua utafiti wa kigenomiki katika idadi ya watu ambao hawajawakilishwa vya kutosha, kwa mfano, kazi kutoka kwa Muungano wa H3Africa [8], Uganda Genome Resource [9], na BioBank Japan [10]. Tofauti na mipango mikubwa, wanaanthropolojia wengi wamejenga tafiti za kigenomiki zinazolenga afya na mageuzi kupitia uhusiano wa muda mrefu na jamii binafsi za kiasili ili kukuza mawasiliano, uaminifu, na uwazi [11–13]. Hata hivyo, jamii za kiasili kote ulimwenguni zinaendelea kuwa miongoni mwa makundi ambayo hayajawakilishwa vizuri katika nyanja yoyote ya utafiti wa kigenomiki [3,4,14].

Hii inatokea kwa sababu jamii za kiasili hazijawakilishwa vyema katika utafiti wa jenetiki kutokana na mwingiliano tata wa mambo, ikiwa ni pamoja na mazoea ya kihistoria ya uchukuaji wa taarifa na unyanyazaji katika nyanja zote za kitabibu na kianthropolojia, kukiwa na kesi mashuhuri nchini Marekani [15,16], New Zealand [17], na kusini mwa Afrika [18]. Hivyo, ukosefu wa uwazi kuhusu malengo ya utafiti, ushiriki mdogo wa jamii, matumizi mabaya ya sampuli, na kushindwa kushughulikia vipaumbele vya jamii vimekwaza ushiriki katika tafiti za kijenetiki zilizopita na kuendelea kuzifanya jamii nyingi kuwa na wasiwasi na kusita kushiriki leo [19]. Katika muktadha huu, jamii kadhaa za kiasili na wasomi wamechapisha mikakati na mapendekezo ya mbinu bora, pamoja na sheria za wazi za ushirikiano [20–23]. Kwa mfano, kutokana na wasiwasi kuhusu ridhaa ya ufahamu, matumizi ya lugha isiyofaa kitamaduni, na uhakiki usiofaa wa maadili wa utafiti uliotumia data zao za kijenetiki [18], watu wa San kusini mwa Afrika walitengeneza kanuni za maadili ya utafiti. Kanuni hizi zimejikita katika misingi mikuu mitano — heshima, uwazi, haki na usawa, utunzaji, na mchakato — na miradi yote ya utafiti hukaguliwa dhidi ya kanuni hizi kabla ya kuidhinishwa [23]. Msingi wa seti hii ya kanuni, pamoja na nyingine zilizotolewa [20,21], umejikita katika ushirikiano wa maana na endelevu na jamii zinazoshiriki wakati wa mchakato wa utafiti. Ushirikiano huu unategemea mawasiliano madhubuti ya malengo ya kisayansi na michakato ili kuhakikisha jamii zinaelewa utafiti unaofanywa, pamoja na faida na mapungufu yake.

Licha ya kukiri uhitaji wa kuwasilisha kwa wazi malengo ya utafiti, taratibu, na matokeo kwa jamii zinazoshiriki, inaweza kuwa vigumu kujadili mada za jenetiki, ambazo mara nyingi ni tata, dhahania, na zimefungamana na maarifa ya asili ya uwanja husika na lugha ya kitaalamu [24]. Kwa mfano, dhana na michakato mingi katika jenetiki haionekani kwa macho, jambo ambalo mara nyingi huzifanya zisiwe rahisi kueleweka kwa hisia za kawaida. Aidha, maneno fulani ya kitaalamu yanayotumika katika Kiingereza, kama vile "DNA", "gene", au "chromosome", yanaweza kukosa maneno yanayolingana moja kwa moja katika lugha nyingine [25]. Hata baadhi ya maneno yanayotumika kuelezea uhusiano wa kifamilia, kama vile "shangazi" au "binamu", yanaweza kukosa tafsiri za moja kwa moja au yanaweza kurejea aina tofauti za uhusiano katika tamaduni na lugha tofauti [26]. Tafiti za kiasi za ujuzi wa jenetiki zinaonyesha zaidi kuwa uelewaji wa dhana msingi za jenetiki unatofautiana sana kati ya watu na unaweza kubadilika baada ya muda [27]. Ili kukabiliana na utata huu, njia moja ambayo wanasayansi wametumia kuwasilisha mada za jenetiki kwa umma na kwa jamii zinazoshiriki ni kupitia matumizi ya picha.

Picha ni mojawapo ya mbinu teule za kufikisha maudhui ya jenetiki kwa sababu zina uwezo wa kutumika kote katika tamaduni na lugha tofauti, zinaonyesha michakato ambayo inaweza isionekane kwa macho, na zinaweza kuwa mwanzo wa kubadilishana dhana [28]. Hata hivyo, hakuna miongozo au mifano iliyokubalika na wengi ya jinsi picha zinapaswa kutengenezwa au kushirikiwa na jamii zinazoshiriki. Ingawa mifano michache imechapishwa—hususan michoro inayotumika kurudisha matokeo [29,30] au kama nyongeza ya ridhaa ya ufahamu [31]—mifano hii inaweza kuwa ya kiufundi sana na kujumuisha kiasi kikubwa cha istilahi za kitaalamu. Zile zinazofikika kwa urahisi zaidi hutegemea mafumbo, ambayo yanaweza kusaidia kutoa taswira ya dhana dhahania kwa kuangazia mambo yanayofanana na matukio yanayofahamika [32]. Kwa mfano, Arango-Isaza et al. walitumia mahindi ya rangi, ambayo ni zao muhimu lenye historia ndefu ya kilimo miongoni mwa jamii za Mapuche waliofanya nazo kazi, kuelezea utofauti wa kijenetiki na urithi [30]. Licha ya maendeleo yanayoendelea katika eneo hili, pengo lililobaki ni kwamba kuna taarifa chache sana katika maandiko kuhusu jinsi picha zinavyotengenezwa, hasa taarifa kuhusu ushirikiano wa mara kwa mara na jamii na maombi pamoja na maoni yao. Matokeo yake, pia inabaki kutoeleweka ikiwa ushiriki na maoni kuhusu nyenzo hizi yanatofautiana kati ya watazamaji, na ni mambo gani mahususi ya kijamii au muktadha ambayo ni muhimu kuzingatiwa.

Hapa, tunashughulikia mapengo haya kwa kufanya utafiti wa kimaelezo na kiasi, unaoendeshwa na jamii ili kuelewa jinsi michoro inavyoweza kuwa na manufaa kwa jamii za kiasili zinazopenda kujifunza zaidi kuhusu jenetiki. Ili kufanya hivyo, tulifanya kazi na makundi mawili ambayo tuna uhusiano wa muda mrefu nayo kupitia tafiti zinazoendelea za kianthropolojia, kigenomiki, na kibiolojia: Orang Asli, watu wa kiasili wa Peninsula ya Malaysia, na Waturkana, wafugaji wa kiasili wa kaskazini-magharibi mwa Kenya. Tulitekeleza mradi huu katika awamu mbili. Kwanza, tuliunda michoro inayojibu maswali yanayoulizwa mara kwa mara kuhusu jenetiki na kukusanya maoni mara kwa mara kutoka kwa wanajamii wa Orang Asli na Turkana kupitia duru kadhaa za kazi ya nyanjani, tukiboresha michoro hiyo kufuatia mapendekezo haya. Ingawa lengo letu la awali lilikuwa kutengeneza picha zinazoweza kutumika kwa ujumla ambazo zinafaa kwa Waturkana na Orang Asli, mchakato huu ulifichua umuhimu wa taswira na mfumo mahususi kwa kila idadi ya watu, na kutupelekea kutoa kipaumbele kwa michoro iliyolengwa kwa jamii badala ya michoro ya jumla (na hivyo kujikita kwa Orang Asli). Kisha, tiliwasilisha michoro ya mwisho mahususi kwa idadi ya watu kwa jamii za Orang Asli na kufanya mahojiano kuhusu michoro hiyo ili kutathmini ufanisi wake. Hatimaye, tulichunguza data ya mahojiano ili kutambua mifumo katika uelewaji na maoni ya washiriki, pamoja na mambo ya kijamii na kimuktadha yanayounda majibu ya washiriki. Kwa ujumla, utafiti huu unajibu maslahi yaliyoelezwa na jamii katika kujifunza zaidi kuhusu jenetiki na kutoa uzoefu kwa watafiti wanaotafuta kushiriki katika juhudi kama hizi za mawasiliano.

**Mbinu**

Idadi ya washiriki

*Orang Asli*

Orang Asli ni watu wa kiasili wa Peninsula ya Malaysia, wakijumuisha chini ya 1% (takriban watu 210,000) ya idadi ya watu nchini humo. Kwa kawaida hugawanywa katika makundi 19 tofauti ya kikabila na kilugha na makundi makuu matatu, yakitofautishwa hasa kwa lugha, muonekano, na mikakati ya kujikimu [33]. Data ya utafiti huu ilikusanywa kutoka vijiji kumi (Picha 1A) ambavyo kimsingi viko katika maeneo kadhaa katika msitu wa mvua wa Peninsula ya Malaysia na kihistoria vimepata ufikiaji mdogo wa miundombinu, ikiwa ni pamoja na elimu rasmi, usafiri, na huduma za afya. Katika vijiji hivyo kuliishi washiriki wa makundi ya kikabila na kilugha ya Batek, Jahai, Temiar, na Semai. Batek na Jahai ni wa kundi dogo la Negrito (Semang), ambao kijadi walikuwa wawindaji na waokotaji wahamaji wanaozungumza lugha za Kiasilia cha Kaskazini, wakati Temiar na Semai ni wa kundi dogo la Senoi, ambao kijadi walifanya kilimo cha kuhama ndani ya mfumo wa maisha wa makazi au nusu-uhamaji na kuzungumza lugha za Kiasilia cha Kati [34].

Katika kipindi cha miaka 50 iliyopita, maendeleo ya haraka ya kijamii na kiuchumi nchini Malaysia yamesababisha mabadiliko makubwa ya mtindo wa maisha kwa Orang Asli, yakichochewa na nguvu kuu mbili: 1) upanuzi wa kilimo cha mashamba makubwa na uchimbaji wa rasilimali asili umemega ardhi za Orang Asli, na 2) programu za serikali zinazohimiza uingiliano katika jamii ya Malaysia zimehamisha Orang Asli wengi kutoka vijiji vya asili kwenda kwenye mipango ya makazi mapya yaliyopangwa. Mradi wa Afya na Mitindo ya Maisha ya Orang Asli (OA HeLP) [35] ni timu ya kimataifa ya wanasayansi, madaktari, na watetezi wa Orang Asli inayozingatia jinsi mabadiliko haya ya mtindo wa maisha yanavyoathiri matokeo ya afya, kwa kutumia aina mbalimbali za maswali, vipimo vya mwili, viashiria vya kibiolojia, na aina za data za kigenomiki [36–39].

Data ilikusanywa kutoka kwa watu wazima wenye umri wa miaka 18 na zaidi waliotoa ridhaa wakati wa safari zilizofanywa kwa ushirikiano na OA HeLP, ikiwa ni pamoja na 1) kliniki zinazotembelea jamii kufanya utafiti na kutoa huduma za afya bila malipo, au 2) safari za kurudisha matokeo kwa jamii ambazo OA HeLP ilishafanya nazo kazi hapo awali. Hata hivyo, ushiriki katika utafiti wa sasa au wa awali wa OA HeLP na kliniki zinazotembea haukuwa sharti la kushiriki katika utafiti huu. Jamii za Orang Asli zilizojumuishwa katika mradi huu zilitambuliwa kupitia mahusiano yaliyopo yaliyojengwa kwa miaka mingi ya kazi ya awali na washiriki wa timu ya OA HeLP. Mchakato wa kuteua washiriki ulijumuisha hatua mbili za jumla. Ruhusa ya kufanya utafiti kwanza ilitafutwa kutoka kwa viongozi wa jamii. Hatua hii ilifuatiwa na mchakato wa ridhaa ya mtu binafsi, ambapo malengo, maswali ya utafiti, na mbinu za mradi huu zilielezwa kwa kina na baada ya hapo ridhaa rasmi ya maandishi ilitolewa.

*Turkana*

Waturkana ni kundi la wafugaji wahamaji wanaoishi katika Bonde la Turkana kaskazini-magharibi mwa Kenya. Utafiti huu ulifanya kazi na watu wa Turkana kutoka vijiji vinne, viwili kati ya hivyo vilikuwa vya mashinani na viwili vilikuwa katika maeneo ya mijini zaidi. Ujenzi unaoendelea wa miundombinu na maendeleo ya haraka ya kiuchumi ya Kenya yamesababisha ukuaji wa vituo kadhaa vya mijini ndani na karibu na ardhi za jadi za Turkana, upanuzi wa masoko madogo, na kuongezeka kwa utegemezi wa viwanda na kilimo. Matokeo yake, Waturkana wengi hawafanyi tena ufugaji wa jadi pekee, badala yake wanategemea biashara, kilimo kidogo, na kuongeza ushiriki katika uchumi wa soko. Mbali na mabadiliko ya kijamii na kiuchumi yanayotokea ndani ya eneo la Turkana, Waturkana wengi wamehamia maeneo yenye miji mikubwa katikati mwa Kenya katika miongo kadhaa iliyopita [40,41].

Mradi wa Afya na Jenetiki wa Turkana (THGP) ni timu ya kimataifa ya wataalamu wa jenetiki na wanabiolojia wanaofanya kazi kuelewa madhara ya kiafya ya mabadiliko haya kwa kutumia maswali yaliyounganishwa, vipimo vya mwili, viashiria vya kibiolojia, na data ya kigenomiki [37,42–44].

Kwa ushirikiano na THGP, mahojiano yasiyo rasmi yalikusanywa kutoka kwa watu wazima wenye umri wa miaka 18 na zaidi waliotoa maoni wakati wa 1) kliniki zinazotembea zinazotembelea jamii kufanya utafiti na kutoa huduma za afya bila malipo, au 2) matukio ya ushirikiano na jamii na uhamasishaji. Ushiriki katika utafiti wa sasa au wa awali wa THGP na kliniki zinazotembea haukuwa sharti la kushiriki katika utafiti huu. Malengo ya utafiti, maswali ya utafiti, na mbinu za mradi huu zilielezwa kwa washiriki katika lugha yao ya asili na watafiti kabla ya ridhaa rasmi ya maandishi kutolewa.

**Muhtasari wa awamu za mradi** Itifaki ya utafiti ilikaguliwa na Bodi ya Mapitio ya Taasisi ya BRANY (itifaki nambari 24-180-734) na ikabainika kuwa imesamehewa. Ukusanyaji wa data ulifanyika katika awamu mbili ili kutengeneza michoro na kutathmini ufanisi wake kama nyenzo ya kuwasilisha taarifa kuhusu utafiti wa jenetiki. Katika awamu ya kwanza (Turkana-2022, Orang Asli-2023, Turkana-2023, Orang Asli-2024; Kielelezo 2), tulitengeneza michoro ya awali kutokana na orodha ya maswali yanayoulizwa mara kwa mara, baada ya hapo mahojiano yasiyo rasmi yalifanyika ili kubaini njia za kuboresha michoro hiyo, kwa kutumia mchanganyiko wa maswali ya majibu huru na maswali ya kuchagua. Katika awamu ya pili (Orang Asli-2025; Kielelezo 2), tulizingatia picha zilizoboreshwa mahususi kwa ajili ya Orang Asli na kufanya mahojiano yaliyopangwa ili kutathmini mwitikio wa washiriki kwa michoro hiyo kwa kutumia maswali ya ndiyo/hapana, majibu mafupi ya nyanja huru, na maswali ya kupanga daraja (tazama Nyenzo za Nyongeza). Hatimaye, tulichanganua data ya mahojiano yaliyokusanywa katika awamu ya pili ili kubaini mifumo katika maoni na majibu ya washiriki.

Uundaji wa awali wa picha (Awamu ya 1)

Ili kusanifu michoro ya awali, tulianza na orodha ya maswali yanayoulizwa mara kwa mara kutoka kwa washiriki katika utafiti tofauti wa muda mrefu wa kianthropolojia na afya wenye malengo sawa na OA HeLP na THGP (yaani, Mradi wa Historia ya Maisha na Afya wa Tsimane [11]). Orodha hii ya maswali ilikusanywa wakati wa kazi ya kurejesha matokeo ya jarida lililochapishwa hivi karibuni kuhusu jenetiki za Tsimane [45]. Tulichagua saba kati ya maswali haya yanayoulizwa mara kwa mara ili kuongoza uundaji wa picha: 1) DNA ni nini?, 2) Je, DNA inaweza kuathiri afya yako?, 3) Wanasayansi wanaweza kujifunza nini kutoka kwa DNA?, 4) Kando na DNA, ni nini kingine unaweza kupata kutoka kwa damu yangu?, 5) Kwa nini wanasayansi wanapenda viashiria vya afya katika damu?, 6) Nini hutokea kwa damu yangu mara tu mnapoichukua?, na 7) Nani ana ufikiaji wa DNA yangu?

Tulitengeneza picha tisa zinazoweza kutumika kwa ujumla ili kuelezea maswali yaliyo hapo juu. Ili kuzingatia viwango tofauti vya uwezo wa kusoma na kuandika, tulipunguza maandishi yaliyoandikwa. Maandishi yote yaliyojumuishwa kwenye michoro iliyojaribiwa Turkana yaliandikwa kwa Kiswahili, na maandishi kwenye michoro iliyojaribiwa na Orang Asli yaliandikwa kwa Kimalay. Ingawa Kiswahili na Kimalay si lugha za asili ndani ya makundi haya, zinafanya kazi kama lugha za mawasiliano ya kikanda (lingua francas) na zinazungumzwa sana katika kila eneo. Tuliomba maoni kutoka kwa wanajamii na wasaidizi wa nyanjani wakati wa misimu miwili ya nyanjani nchini Kenya (Turkana-2022, Turkana-2023) na misimu miwili ya nyanjani nchini Malaysia (Orang Asli-2023 na Orang Asli-2024). Tulitumia mbinu ya kurudia ili kujumuisha maoni ya jamii, tukiboresha picha wakati wa kila msimu wa nyanjani (Kielelezo 1C). Hatimaye, matoleo mawili ya michoro yalizalishwa -- toleo moja la jumla linalofaa kwa mazingira mengi (tazama <https://github.com/audreyarner/genetic_illustrations>), na toleo moja mahususi kwa Orang Asli (<https://github.com/tcmccabe/OrangAsliHealthIllustrations> ).

Uwasilishaji wa michoro (Awamu ya 1 na Awamu ya 2)

Katika Awamu ya 1, tulijaribu usambazaji wa michoro kwa kutumia mifumo mingi—ikiwa ni pamoja na mawasilisho ya slaidi ya mubashara, video zilizorekodiwa mapema, utazamaji wa kutumia tableti, na nyenzo za majadiliano zilizochapishwa—ili kuboresha kile kilichokuwa na manufaa zaidi kwa washiriki na ambacho kingeweza kutekelezwa kwa uhakika, ikizingatiwa kwamba baadhi ya maeneo hayana ufikiaji thabiti wa umeme, wifi, au huduma za simu. Sawa na michoro yenyewe, uwasilishaji wa michoro uliboreshwa kwa njia ya kurudia, ukijumuisha maoni ili kubaini mbinu yenye manufaa zaidi.

Michoro hiyo iliwasilishwa kwa mara ya kwanza katika tukio la ushirikiano wa jamii ya Turkana lililofanyika Oktoba 2022 (Turkana-2022). Iliwasilishwa kama hotuba ya mubashara, ambapo msaidizi wa utafiti wa THGP aliwasilisha nyenzo hiyo kwa Kiswahili. Kila mchoro ulionyeshwa kwenye slaidi ya PowerPoint na kuonyeshwa kupitia projekta. Baadaye katika msimu huo huo (Turkana-2022), tiliwasilisha seti ya picha zilizorekebishwa katika wasilisho la slaidi la dakika 15 ambalo lilisimuliwa kwa Kiswahili na kuonyeshwa kwa watu binafsi katika vijiji vitatu wakati wa kliniki za afya zinazotembea. Muundo wa video ulihakikisha uwasilishaji thabiti kila wakati. Muundo wa video pia uliwasilishwa katika Tamasha la Kitamaduni la Turkana la 2023, ambapo washiriki wa timu ya THGP waliandaa banda lililoangazia shughuli zao pana za utafiti na kuwasilisha michoro (Turkana-2023).

Tulipia tuliwasilisha picha hizo katika muundo wa video ya dakika 15 kwa jamii za Orang Asli (Orang Asli-2023). Katika kisa hiki, video ilirekodiwa mapema ikiwa na maelezo ya Kimalay na kuonyeshwa kwa wanajamii katika mikusanyiko mikubwa ya jamii (~watu 20) kwa kutumia projekta au vikundi vidogo (watu 2-5) kwenye tableti (Kielelezo 1B).

Kulingana na maoni, tuliboresha muundo wa uwasilishaji hivi kwamba wasaidizi wa utafiti wa OA HeLP waliongoza mawasilisho madogo ya majadiliano ya picha zilizochapishwa, wakielezea kila picha kwa makundi ya watu mmoja hadi sita (Orang Asli-2024). Kila mchoro ulichapishwa na kuwekwa kwenye plastiki (laminated) kwenye karatasi ya A4. Ingawa taarifa zilizotolewa zilitofautiana kidogo kati ya vipindi, muundo huu uliruhusu majadiliano ya mwingiliano na ushiriki wa vitendo na nyenzo hizo. Tulishirikisha sehemu pana ya wanajamii katika majadiliano haya, wakiwemo Tok Batin (wakuu wa vijiji), walimu, wazee, na vijana ambao walikuwa wamemaliza masomo ya sekondari hivi karibuni. Ili kuhakikisha upatikanaji nje ya mazingira ya kidijitali na kama rasilimali ya baadaye, pia tulitengeneza vijitabu vilivyochapishwa vyenye maudhui yale yale (tazama Taarifa za Nyongeza, Orang Asli-2024).

Katika Awamu ya 2 (Orang Asli-2025), michoro ya mwisho ilionyeshwa wakati wa uwasilishaji wa mubashara katika mazingira ya kikundi cha wastani hadi kikubwa ili kutathmini ufanisi na kukusanya maoni ya washiriki. Sawa na miundo ya awali, uwasilishaji huu ulitumia slaidi za PowerPoint (wakati umeme ulipopatikana) au matoleo yaliyochapishwa na kuwekwa kwenye plastiki kwa ajili ya washiriki kutazama [2][3]. Mawasilisho haya yalitolewa mubashara katika Kimalay na msaidizi wa utafiti wa OA HeLP, jambo ambalo liliruhusu mwingiliano na kukatizwa ikiwa watazamaji walikuwa na maswali. Msaidizi wa utafiti aliyehusika katika Awamu ya 2 hakuwa na asili katika masuala ya jenetiki, bali alikuwa amejadili maelezo ya picha na mtafiti mwenye asili katika fani hiyo ili kuboresha usemi na uelewaji. Mawasilisho ya mwisho yalidumu takriban dakika 12. Uwasilishaji huo ulitolewa katika jamii sita za Orang Asli, kukiwa na washiriki kati ya 20 na 70 waliohudhuria kila kipindi. Tena tulitoa vijitabu vyenye maudhui yale yale kwa washiriki kwa ajili ya kumbukumbu ya baadaye.

Mahojiano (Awamu ya 1 na Awamu ya 2)

Katika Awamu ya 1, tulifanya mahojiano mafupi, yasiyo rasmi wakati wa misimu minne ya nyanjani (Turkana-2022, Orang Asli-2023, Turkana-2023, Orang Asli-2024) ili kutathmini jinsi michoro, uwasilishaji wake, na mahojiano yenyewe yanavyoweza kuboreshwa ili kukidhi mahitaji ya jamii vizuri zaidi. Mahojiano yalifanywa kwa Kimalay na washiriki wa Orang Asli au Kiswahili na washiriki wa Turkana, na watu walikuwa huru kujibu maswali yoyote waliyotaka. Maswali yalijumuisha vipengele vya majibu huru kuhusu kile ambacho watu walipenda zaidi na kidogo kuhusu picha hizo, pamoja na maswali ya kupima maarifa yaliyopatikana yakitathmini uelewaji wa baadhi ya dhana za jenetiki zilizochorwa.

Katika Awamu ya 2, tulifanya mahojiano rasmi ya muundo mfupi na washiriki wa Orang Asli ili kukusanya data ya ubora na wingi (Orang Asli-2025). Mahojiano haya yalilenga maeneo makuu manne: taarifa za kidemograsia, maarifa ya awali kabla ya kutazama wasilisho, maoni kuhusu michoro, na uwezeshaji wa maarifa unaodhaniwa (tazama Taarifa za Nyongeza). Maswali kadhaa yaliboreshwa kutoka yale yaliyojaribiwa wakati wa misimu ya nyanjani ya mapema. Mahojiano yote yalifanywa kwa Kimalay na wasaidizi wa utafiti wa OA HeLP wa eneo hilo na yalidumu takriban dakika 10-15. Kwa jumla, washiriki 92 katika vijiji sita walikamilisha mahojiano rasmi (Jedwali la SI 2).

Uchambuzi wa kimaudhui wa majibu ya maswali ya nyanja huru (Awamu ya 2)

Ili kutambua mawazo mapana yanayotokana na majibu ya maswali matatu ya mahojiano ya nyanja huru, tulifanya uchambuzi wa kimaudhui wa mchakato wa kuzalisha [46] wa washiriki, tukitumia uchambuzi wa kimaudhui kwa majibu ya kila swali kando. Hasa, watafiti wawili (A.M.A. & A.S.) walifanya usimbaji (coding) wa majibu yote kwa kila swali kwa njia ya kujiendesha wakitumia programu ya MAXQDA toleo la 26 ili kutambua dhana na mifumo inayojirudia. Jibu fulani linaweza kuwa na vishazi vingi vinavyowasilisha mawazo tofauti. Kwa hivyo, kitengo cha uchambuzi katika usimbaji kilikuwa kishazi kilichounganishwa na wazo. Usimbaji wa majibu ya kila swali la mahojiano ulijadiliwa, ambapo watafiti walipitia kwa mfumo maalum tafsiri za kodi na matumizi yake, huku tofauti zikitatuliwa kwa makubaliano. Asilimia ya makubaliano ilihesabiwa ili kutathmini uthabiti wa kodi kwa kila msahihishaji, ambayo ilianzia 86% hadi 100% ya makubaliano (Jedwali la SI 3). Watafiti walitambua kwa kujitegemea mada za ngazi ya juu zilizojitokeza kutoka kwa kodi zilizotambuliwa, ikifuatiwa na majadiliano ya kufafanua na kuboresha mfumo wa kimaudhui, maneno, na maelezo kwa kila mada.

Uchambuzi wa takwimu wa data ya mahojiano (Awamu ya 2)

Kwanza, tulitumia mifumo ya binomial ili kupima ikiwa idadi ya watu wanaojibu kwa ushirikiano (ndiyo) kwa kila swali la ndiyo/hapana ilitofautiana na ile inayotarajiwa kwa nasibu, tukilinganisha mifumo kando kwa kila swali. Tulirekebisha majaribio ya nadharia nyingi kwa kutumia kiwango cha ugunduzi wa uongo cha Benjamini-Hochberg [47]. Pia tulihesabu tumbo la uwiano la Pearson ili kutathmini jinsi majibu ya kila swali yanavyobadilika kwa pamoja kati ya watu.

Pili, tulipima ikiwa majibu ya maswali katika mahojiano yetu yanaweza kuunganishwa katika mihimili ya tofauti (kwa mfano, ikiwa makundi ya watu walielekea kujibu maswali fulani kwa namna inayofanana). Ili kufanya hivyo, tulitumia uchambuzi wa mawasiliano mengi (MCA), aina ya uchambuzi wa sababu za uchunguzi ulioundwa ili kupunguza mwelekeo wa data ya kategoria [48]. MCA yetu ilijumuisha seti ya maswali nane ya ndiyo/hapana yaliyobadilishwa kuwa muundo wa Boolean (kweli/uongo). Watu wawili waliondolewa kutokana na jibu moja au zaidi kukosekana, na kusababisha jumla ya watu 90 kwa uchambuzi huu. MCA ilifanyika kwa kutumia kifurushi cha FactoMineR katika R kwa vigezo vya kawaida [49]. Vipimo viwili vya kwanza vilihifadhiwa kulingana na hali yao ya kutosha (35.3% na 18.9% mtawalia; tazama SI Kielelezo 1) na uwezo wa kutafsiriwa.

Kisha tulipima ikiwa kuratibu za mtu binafsi kwenye vipimo vya MCA 1 na 2 viliathiriwa na sababu zozote za kijamii na kidemograsia, ambazo ni jinsia, umri, elimu ya juu (iliyopewa kodi kama kigezo cha mstari ambapo 0 inawakilisha kutokuwa na elimu rasmi, 1 inawakilisha sehemu ya elimu ya msingi, 2 inawakilisha sehemu ya elimu ya sekondari, na 3 inawakilisha sehemu ya elimu ya chuo kikuu), na "hali ya mijini". Hapa, tulitumia "alama ya hali ya mijini" inayozingatia eneo ambayo inakamata ufikiaji wa rasilimali za viwandani, zinazozingatia soko zinazopatikana katika jamii nzima (kwa mfano, ufikiaji wa umeme, maji taka, elimu rasmi; tazama Maandishi ya Nyongeza kwa ajili ya kuzalisha alama ya hali ya mijini). Alama hii ilipendekezwa kwanza na Novak et al [50] na imekuwa ikijaribiwa hapo awali kwa Orang Asli [36]. Tulitumia mifumo ya mstari ikijumuisha jinsia, umri, kiwango cha juu cha elimu, na alama ya hali ya mijini ili kutabiri vipimo vya MCA 1 na 2 katika mifumo tofauti [47]. Pia tuliendesha mifumo ya ufuatiliaji ambapo kiwango cha juu cha elimu kiliwekwa kama kigezo cha binary cha kutokuwa na elimu rasmi (iliyopewa kodi kama 0) dhidi ya kiwango chochote cha elimu rasmi (iliyopewa kodi kama 1).

Hatimaye, tulitumia mifumo ya mstari kuchanganua ikiwa sababu za kidemograsia na nyinginezo ziliathiri jibu la kila swali la ndiyo/hapana. Kwa kila swali kando, tulilinganisha mfumo wa binomial ambapo jibu (ndiyo/hapana) lilitabiriwa kwa pamoja na umri, jinsia, kiwango cha juu cha shule, au alama ya hali ya mijini. Tulirekebisha tena kwa ajili ya majaribio ya nadharia nyingi kwa kutumia mbinu ya FDR. Sawa na hapo juu, tuliendesha mifumo ya ufuatiliaji tukibadilisha kiwango cha juu cha elimu na kigezo cha binary cha kutokuwa na elimu rasmi dhidi ya kiwango chochote cha elimu rasmi. Uchambuzi wote ulifanyika kwa kutumia lugha ya kompyuta ya R na RStudio (toleo la 4.2.1).

**Matokeo**

Tulitengeneza mfululizo wa michoro ili kushughulikia maswali yanayoulizwa mara kwa mara kuhusu jenetiki. Toleo la jumla na toleo mahususi kwa Orang Asli la michoro hiyo yanapatikana katika Taarifa za Nyongeza, GitHub (<https://github.com/audreyarner/genetic_illustrations>, <https://github.com/tcmccabe/OrangAsliHealthIllustrations>) na pia yanapatikana kwenye tovuti ya mradi wa OA HeLP [4][5][6][7] (<https://www.orangaslihealth.org/>). Katika maandishi yafuatayo, tunaelezea: Awamu ya 1, ambayo ilijumuisha maendeleo na uboreshaji wa mara kwa mara wa michoro; na Awamu ya 2, ambayo ilijumuisha uwasilishaji wa toleo la mwisho la michoro na tathmini za ubora na wingi za ufanisi wake na vichocheo vya ushiriki.

**Awamu ya 1: Maendeleo ya mara kwa mara ya michoro hutegemea maoni ya jamii**

Kwa kuzingatia umuhimu wa rasilimali ya jenetiki inayoweza kutumika kwa ujumla, duru yetu ya kwanza ya picha za majaribio ilionyesha watu, vitu, na mazingira ambayo hayakuwa mahususi kwa eneo lolote la kijiografia (Kielelezo 1C). Kwa mfano, tulitumia mchoro wa umbo la binadamu uliorahisishwa bila sifa zozote zinazotambulika ili kuongeza uwezekano wa watu kujihusisha nayo katika mazingira tofauti, kulingana na picha za jenetiki zilizotumiwa katika machapisho ya awali [29,30]. Mada kuu katika maoni ya mapema (Turkana-2022) ilikuwa hamu ya picha zenye uhalisia zaidi. Kwa kujibu, tulirekebisha michoro ili kujumuisha maelezo zaidi ya kuona na picha zisizo za kidhahania za watu, ikijumuisha aina mbalimbali za rangi za ngozi (Kielelezo 1D). Maoni kuhusu michoro iliyorekebishwa kwa ujumla yalikuwa chanya; hata hivyo, watazamaji waliona mbinu ya usambazaji wa michoro (video; Turkana-2023, Orang Asli 2023), kuwa ndefu sana na isiyo na mwingiliano wa kutosha. Kwa hivyo, washiriki walipendekeza kujumuishwa kwa vipengele vyenye mjongeo zaidi kama vile uhuishaji. Sawa na maoni kutoka Turkana, watazamaji wa Orang Asli walidokeza kuwa video ilikuwa ndefu sana, huku watazamaji wakiripoti kuwa mtindo huu wa uwasilishaji haukuwa na mwingiliano wa kutosha (Orang Asli-2023). Tofauti na maoni kutoka Turkana, Orang Asli waliripoti kutaka uwasilishaji ujumuishe picha ambazo ziliendana zaidi na mazingira yao ya ndani na mahususi kwa maisha yao.

Kulingana na maoni haya ya jamii, tulipea kipaumbele kufupisha wasilisho, kulifanya wasilisho liwe na mwingiliano zaidi, na kujumuisha vipengele mahususi vya Orang Asli katika michoro kwa ajili ya majaribio ya ziada (Kielelezo 1E, Orang Asli-2024). Ili kufupisha wasilisho tuliondoa moja ya picha (ambayo ilijibu swali "Nini hutokea kwa damu yangu mara tu mnapoichukua") ambayo ilikuwa inajirudia zaidi. Kwa kila picha, tulijumuisha picha zinazohusika zilizopigwa katika jamii za Orang Asli, na pia tukaongeza mandhari ya msitu wa mvua kwa kila mchoro. Pendekezo la kawaida zaidi tulilopokea katika hatua hii lilikuwa kujumuisha mifano na picha zaidi mahususi kwa Orang Asli, pamoja na mifano ya kanuni za jenetiki ambazo wanajamii wangekuwa wanazifahamu zaidi. Ingawa lengo letu la awali lilikuwa kutoa rasilimali inayoweza kutumika kwa ujumla inayofaa kwa watu wengi, maoni haya yalichochea mabadiliko kuelekea kutengeneza toleo mahususi la Orang Asli kama seti kuu ya michoro.

Maoni pia yalichangia maendeleo ya maswali yetu ya mahojiano. Wakati wa mahojiano ya mapema ya majaribio (Turkana-2022, Orang Asli-2023), baadhi ya wanajamii walibaini kuwa ilionekana kama kufanya mtihani, jambo ambalo walisema halikufurahisha. Kwa sababu lengo letu lilikuwa kutathmini jinsi michoro ilivyokuwa na ufanisi kwa uwezeshaji wa maarifa badala ya upataji wa maarifa kwa viwango vya Kimagharibi, tuliondoa vipengele vyenye majibu ya uhakika ya "sahihi" au "si sahihi". Zaidi ya hayo, tulipunguza idadi ya maswali ya nyanja huru yaliyojumuishwa katika mahojiano ya mwisho, kwani tuligundua kuwa watu walikuwa na wakati mgumu kuelezea baadhi ya dhana kwa maneno bila vidokezo. Kwa jumla, maoni ya jamii yalikuwa ya kujenga mara kwa mara, yakisisitiza shukrani kwa mbinu ya picha na mazungumzo na kuonekana kwa kuongezeka kwa umuhimu wa michoro hiyo kwa uzoefu wao wenyewe.

**Awamu ya 2: Maudhui ya mwisho na uwasilishaji ulilenga picha mahususi za idadi ya watu**

Michoro iliyokamilika iliundwa kulingana na maswali sita yanayoulizwa mara kwa mara kuhusu jenetiki (Kielelezo 3; Jedwali la SI 1; Orang Asli-2025). Ikiongozwa na mapendekezo ya jamii, toleo hili mahususi la Orang-Asli lilijumuisha mifano inayotambulika ya ndani ya jenetiki. Kwa mfano, muundo wa nywele -- ambao unatofautiana sana ndani ya vikundi vya kijamii na lugha vya Orang Asli -- ulitumiwa kuelezea urithi (Kielelezo 3B), ukibadilisha urefu ambao unaonyesha tofauti ndogo inayoonekana katika eneo hilo. Zaidi ya hayo, tulitumia durian, tunda maarufu nchini Malaysia ambalo lina aina nyingi zinazotambulika kwa urahisi zinazotofautiana katika mwonekano, muundo, na ladha, kuelezea utofauti wa kijenetiki na athari ambazo mazingira yanaweza kuwa nazo kwenye sifa (Kielelezo 3C). Kwa sababu kilimo cha kiwango kidogo na mwingiliano wa karibu na mimea iliyolimwa na ya mwituni ni sehemu ya maisha ya kila siku kwa jamii nyingi za Orang Asli, mfano huu ulitumia maarifa ya pamoja ya uzoefu kufanya utofauti wa kijenetiki ufikike kwa urahisi zaidi. Marekebisho sawa ya msingi ya ndani yalifanywa katika michoro yote, ikiwa ni pamoja na kuonyesha watu katika mavazi ya kitamaduni na nyumba.

Awamu ya 2: Michoro iliyoripotiwa kuwa na manufaa na washiriki

Ili kuelewa ikiwa michoro hiyo ilisaidia katika kufikisha dhana za jenetiki, tulifanya mahojiano rasmi na watu 92 wa Orang Asli (Jedwali la Nyongeza 2). Kwa jumla, washiriki 85 (92%) waliripoti kutaka kujua zaidi kuhusu utafiti wa jenetiki, na washiriki 44 (48%) waliripoti kuwa waliamini kabla ya kuona michoro hiyo kuwa kuna taarifa zinazohusiana na afya katika damu yao. Washiriki waliojibu kwa ushirikiano (ndiyo) kwa swali la pili waliulizwa swali la ufuatiliaji kuhusu aina ya taarifa walizoamini zingekuwepo. Watu tisa hawakuwa na jibu mahususi. Kwa wale waliojibu, tulitumia uchambuzi wa kimaudhui ili kubaini mada kuu mbili zilizotokana na data (SI Kielelezo 2). Kwanza, washiriki walielezea ujuzi wa viashiria vinavyopimika vinavyotokana na damu, mara nyingi wakirejelea alama mahususi za kibaolojia au vipimo (kwa mfano, "damu ina sukari ndani yake"). Pili, washiriki walielezea ujuzi kwamba damu inaweza kutumika kutathmini hali ya matibabu, ikionyesha tafsiri pana za afya (kwa mfano, "magonjwa na afya").

*Uchambuzi wa Ubora: Mapendeleo ya michoro yanaendana na ujuzi wa mazingira, huku picha za kiufundi zikichanganya zaidi*

Kisha tiliuliza maswali kuhusu michoro ili kuelewa mapendeleo ya washiriki na maeneo yenye mkanganyiko. Asilimia kubwa zaidi ya washiriki (38%) waliripoti Mchoro B kama picha waliyoipenda zaidi (Kielelezo 3B). Hii ni picha ambayo Orang Asli huenda wangekuwa wanaifahamu zaidi, ikionyesha urithi wa muundo wa nywele -- sifa yenye tofauti inayoonekana miongoni mwa Orang Asli -- ikionyesha watu wanaoishi na familia zao katika nyumba za kitamaduni za mianzi zilizoko katika mazingira ya msitu wa mvua.

Ili kutathmini rasmi kwa nini picha fulani zilipendelewa, tulifanya uchambuzi wa kimaudhui wa maelezo ya nyanja huru ya washiriki kwa picha walizozipenda zaidi (SI Kielelezo 3). Mada ya kawaida zaidi ilikuwa upendeleo wa picha zinazohusiana na utambulisho, huku washiriki wakirejelea mara kwa mara mazingira yanayotambulika na uzoefu wa maisha, wakibainisha, kwa mfano, kwamba mchoro huo "ni sawa na shughuli zangu za kila siku, kama kucheza takraw". Washiriki pia walisisitiza kupendezwa na dhana za jenetiki, wakielezea kuwa walipenda baadhi ya picha kwa sababu "walipenda kujua kwamba kila mtu ana DNA yake mwenyewe". Makundi madogo ya washiriki walielezea upendeleo kwa vipengele vya kuona na urembo (kwa mfano, "picha ni nzuri na ya kifahari"), picha zinazohusiana na afya (kwa mfano, "kwa sababu picha inaonyesha jinsi unavyoweza kuwa na afya"), na michoro inayonyesha kuwa kuna faida za kiafya zinazohusiana na mtindo wa maisha (kwa mfano, "mtindo wa maisha wa afya wa kijijini").

Pia tuliuliza ni picha gani, ikiwa zipo, zilizokuwa zinachanganya kwa watazamaji (Kielelezo 4B); 85% ya washiriki waliripoti angalau picha moja kuwa inachanganya (wastani = picha 1.8 zinazochanganya). Kwa mfano, picha iliyoripotiwa mara nyingi kuwa inachanganya (iliyochaguliwa katika 30% ya majibu yote ya picha, huku washiriki wakiwa na uwezo wa kuchagua picha nyingi) ilikuwa Mchoro G, ambao unaonyesha tofauti za DNA katika jamii nyingine za asili. Picha hii ilikuwa ya kiufundi zaidi; hata hivyo, ilihifadhiwa katika seti ya mwisho ya michoro kutokana na maoni kutoka Awamu ya 1 ya kujumuisha taarifa kuhusu idadi nyingine ya watu asilia duniani kote.

Ili kuelewa mada zenye maslahi ya kudumu, tuliuliza ni picha gani, ikiwa zipo, washiriki wangetaka kujifunza zaidi kuzihusu. Washiriki wengi waliripoti kutaka kujifunza zaidi kuhusu angalau picha moja (wastani = picha 1.4). Maslahi yalisambazwa kwa usawa katika michoro tofauti (Kielelezo 4C). Kama ufuatiliaji, tuliuliza ni mada gani za jumla washiriki wangependa kujifunza zaidi kuzihusu (Jedwali la SI 4). Washiriki wengi walielezea nia ya kujifunza zaidi kuhusu afya na magonjwa (49% ya watu) na uhusiano wa kifamilia (46% ya watu). Idadi ndogo ya washiriki walichagua "nyingine", kimsingi wakiibua maswali kuhusu aina ya damu.

Hatimaye, tuliuliza washiriki jambo gani moja walilojifunza kutoka kwa michoro hiyo. Tulitumia tena uchambuzi wa kimaudhui wa majibu mafupi ya nyanja huru, ambayo yalifunua mada kuu nne (SI Kielelezo 4). Mada ya kawaida zaidi ilionyesha kuongezeka kwa uelewaji wa jukumu la DNA na damu mwilini, huku washiriki wakielezea ufahamu mpya kwamba damu ina taarifa za kibaolojia na kwamba DNA huathiri sifa za mwili na afya. Kwa mfano, baadhi ya washiriki walibainisha "kila mtu ana DNA yake mwenyewe" na "mabadiliko ya DNA yanaweza kuathiri afya". Mada ya pili ilihusisha sababu zinazochangia afya na ustawi. Majibu yalirejelea kujifunza, kwa mfano, kwamba "damu ina taarifa za afya." Mada ya tatu ilihusu utambuzi wa tofauti za kijenetiki kati ya watu binafsi na idadi ya watu. Hatimaye, mada ndogo lakini muhimu ilionyesha ufahamu wa upungufu wa maarifa, huku washiriki wakitambua ugumu wa kuelezea dhana mahususi waliyojifunza au wakibainisha kuwa wanataka kujifunza zaidi katika siku zijazo.

*Uchambuzi wa wingi: Michoro ya jenetiki ilivutia kwa upana na kuboresha uelewaji, na ushiriki ulionyesha mabadiliko kiasi kulingana na elimu, jinsia, na hali ya mijini*

Tulijaribu kuelewa ufanisi wa michoro hiyo kwa mahitaji ya washiriki kwa kuuliza maswali manane ya ndiyo/hapana yanayotathmini nia na maarifa yaliyopatikana yaliyoripotiwa na washiriki wenyewe. Maswali yote yalijibiwa kwa ushirikiano (ndiyo) zaidi ya ilivyotarajiwa kwa nasibu ($FDR < 0.05$), ikionyesha kuwa washiriki waliona michoro hiyo kuwa inavutia, inaeleweka, na yenye kuelimisha zaidi kuliko sivyo (Kielelezo 6A, Jedwali la SI 5). Kisha tulitathmini uwiano kati ya majibu (Kielelezo 6B). Majibu yalionyesha uthabiti wa ndani, huku vipimo vya uelewaji na ushiriki vilivyoripotiwa vikiwa na uwiano chanya (kwa mfano, "Ningeitazama tena picha hizi" na "Ningependekeza kwa rafiki"). Kuripoti kuwa angalau mchoro mmoja ulikuwa mgumu kueleweka kulikuwa na uwiano hasi na karibu maswali mengine yote, hasa maswali yanayohusiana na ushiriki na mapendekezo kwa wengine (wastani wa Pearson $r = -0.2$).

Ili kuchunguza zaidi uwiano katika majibu ya washiriki, tulifanya uchambuzi wa mawasiliano mengi (MCA) tukitumia maswali yote manane ya ndiyo/hapana. Tulibaini kuwa vipimo viwili vilichangia sehemu kubwa ya tofauti (kipimo cha 1: 35.3%, kipimo cha 2: 18.9%; SI Kielelezo 1). Kipimo cha kwanza kilionekana kuakisi zaidi nia na ushiriki, kikiwa na uzito mkubwa kwa vipengele kama vile "Ningependekeza michoro hii kwa rafiki" na "Nataka kujifunza zaidi" (Kielelezo 6C). Kipimo cha pili kilichochewa na maswali yanayohusiana na uelewaji na uwazi, kikiwa na nguvu zaidi kwenye maswali kama vile "michoro ilinisaidia kuelewa zaidi kuhusu jenetiki" na "naelewa kwa nini wanasayansi wangetaka kusoma DNA" (Kielelezo 6D). Kisha tulinganisha mahusiano kati ya alama za watu binafsi kwenye vipimo viwili vya kwanza vya MCA na vichocheo vinne: umri, jinsia, kiwango cha juu cha elimu kilichofikiwa, na hali ya mijini. Ingawa hakuna kichocheo kilichobaki kuwa muhimu baada ya marekebisho ya majaribio mengi ya nadharia, kipimo cha 2 kilihusishwa kwa jina na alama ya hali ya mijini ($p=0.042$) (Jedwali la SI 6).

Hatimaye, tulilinganisha kila swali la mahojiano kama kigezo cha umri, jinsia, kiwango cha juu cha elimu, na hali ya mijini (Vielelezo 6E, Jedwali la SI 7). Wakati hakuna vichocheo vilivyobaki kuwa muhimu baada ya marekebisho ya majaribio mengi ya nadharia, tuligundua kuwa kiwango cha juu cha elimu kilionyesha mahusiano thabiti zaidi, kikifikia kiwango cha jina $P < 0.05$ kwa maswali matatu. Kiwango cha juu cha elimu kilihusishwa na majibu ya "ndiyo" kwa maswali yote matatu (Kielelezo 6F). Zaidi ya hayo, tuligundua kuwa watu wenye hali ya chini ya mijini walikuwa na uwezekano mkubwa wa kuripoti kuwa michoro hiyo iliwasaidia kuelewa kwa nini watafiti wanataka kusoma DNA ($P=0.03$; Jedwali la SI 7), huku watu wenye hali ya chini ya mijini walikuwa na uwezekano mkubwa wa kuripoti kuwa wangeweza kuelezea michoro hiyo kwa rafiki ($P=0.003$; Jedwali la SI 7). Hatimaye, wanaume walikuwa na uwezekano mkubwa kuliko wanawake kuripoti kuwa baadhi ya michoro ilikuwa migumu kueleweka ($P=0.01$; Jedwali la SI 7). Tuligundua kuwa matokeo yalikuwa sawa sana wakati wa kutumia kigezo cha binary cha kutokuwa na elimu dhidi ya kuwa na elimu yoyote rasmi (Jedwali la SI 8). Pamoja, mifumo hii inadokeza kuwa kukutana na elimu rasmi hapo awali na dhana za jenetiki, ambazo hutofautiana kimfumo na hali ya mijini, kunaweza kuathiri jinsi watu wanavyotafsiri na kujihusisha na nyenzo za mawasiliano ya jenetiki.

**Majadiliano**

Mawasiliano madhubuti ya taarifa za jenetiki ni muhimu sana kwa ushirikiano wa kimaadili wa utafiti [20, 23]. Hata hivyo, mara nyingi dhana za jenetiki ni za kidhahania, za kiufundi, na ni changamoto kuziwasilisha katika mazingira tofauti ya kilugha na kitamaduni. Mbinu za kielelezo zinaweza kuziba pengo hili kwa kuunganisha dhana zisizoshikika na mifano halisi inayonekana. Wakati mifano michache ya michoro [29–31] na video [51, 52] zinazoelezea dhana za jenetiki kwa jamii zinazoshiriki imechapishwa, kuna taarifa chache kuhusu jinsi taswira hizi zinavyoundwa au jinsi zinavyopokelewa. Hapa, tulitumia mchakato wa kurudia-rudia unaozingatia jamii ili kuonyesha kwamba michoro inaweza kutumika kama zana madhubuti za kuwasiliana kuhusu utafiti wa jenetiki na jamii za asili, lakini ushiriki na uelewaji huathiriwa na mambo ya kidemograsia na muktadha.

Kwanza, tuligundua kuwa maendeleo ya kurudia-rudia yanayoongozwa na jamii yalikuwa muhimu kwa ajili ya kuzalisha michoro iliyoendana na vipaumbele vya washiriki. Picha za awali tulizounda (Kielelezo 1C) zilikuwa tofauti sana na matoleo ya mwisho (Kielelezo 1F), kukiwa na duru nyingi za marekebisho zilizotokana na maoni ya jamii. Mchakato huu unaenda sambamba na mbinu za utafiti shirikishi zinazosisitiza uundaji wa pamoja na usikivu kwa watumiaji badala ya uwasilishaji wa maarifa wa upande mmoja. Hakika, kazi za awali katika mawasiliano ya sayansi na utafiti shirikishi unaozingatia jamii zimeonyesha kuwa maendeleo ya kurudia-rudia yanaboresha umuhimu, imani, na ushiriki, hasa wakati wa kuwasiliana mada tata au nyeti [53, 54]. Inashangaza kwamba, mchakato huu wa kurudia ulifunua kwamba vipaumbele vya washiriki si mara zote vililenga kurahisisha mambo kupita kiasi au uwazi wa papo hapo. Kwa mfano, ingawa washiriki wengi waliripoti angalau mchoro mmoja kuwa unachanganya (Kielelezo 4C), picha iliyotambuliwa mara nyingi zaidi (Kielelezo 3E, inayoonyesha tofauti za kijenetiki miongoni mwa watu asilia duniani kote) ilihifadhiwa makusudi kufuatia maoni ya washiriki yaliyosisitiza umuhimu wa kuelewa jinsi jamii za ndani zinavyoingia katika muktadha mpana wa watu asilia.

Pili, tuligundua kuwa washiriki walipendelea sana michoro iliyoakisi watu wanaowafahamu, mazingira, na uzoefu wa maisha. Wakati wa awamu za mapema za uundaji, washiriki wa Turkana na Orang Asli walielezea kutoridhika na michoro iliyotumia maumbo ya jumla ya binadamu au iliyojaribu kuonyesha utofauti kupitia rangi mbalimbali za ngozi; badala yake, washiriki walitaka kuona watu wanaofanana nao na muktadha unaoakisi jamii zao wenyewe. Ugunduzi huu unaendana na kazi za awali zinazoonyesha kuwa mifano inayozingatia uzoefu wa pamoja hurahisisha uelewaji wa dhana za kibayolojia zisizoshikika. Kwa mfano, usimulizi wa hadithi kidijitali katika jamii za wenyeji wa Alaska umeonyeshwa kuwa mbinu inayozingatia utamaduni na inayovutia katika mawasiliano ya sayansi, hasa wakati masimulizi hayo yanajikita katika maarifa na uzoefu wa ndani [55].

Kwa upana zaidi, ugunduzi huu unaakisi mifumo inayozingatiwa katika mifumo ya utawala wa kimaadili: wakati misingi ya kimataifa kama vile Azimio la Umoja wa Mataifa kuhusu Haki za Watu wa Asili (UNDRIP) na kanuni za CARE [56,57] zinatoa mwongozo muhimu, utekelezaji wake madhubuti unahitaji umakini kwa historia, utamaduni, na vipaumbele mahususi vya kila jamii ya asili, ambavyo si sawa kote [23,58,59].

Hatimaye, tofauti tulizoziona katika ushiriki na uelewaji wa michoro hiyo inasisitiza zaidi hitaji la kuacha mbinu ya jumla ya "saizi moja inatosha wote". Ingawa washiriki kwa ujumla walionyesha nia kubwa ya kujifunza kuhusu jenetiki, ushiriki thabiti, na hisia ya kuongezeka kwa uelewaji, uchambuzi wetu wa wingi ulionyesha uhusiano mdogo lakini thabiti kati ya majibu na hali ya mijini, elimu, umri, na jinsia.

Kiwango cha elimu hasa kiliibuka kama kichocheo kikuu, kikionyesha uwezekano wa tofauti za kukutana na mada zinazohusiana na jenetiki. Watu wenye elimu rasmi zaidi walikuwa na uwezekano mkubwa wa kuripoti kuwa wangeweza kuelezea michoro hiyo kwa rafiki, jambo linaloakisi uwezekano wa kufahamiana zaidi na mada hizi hapo awali, lakini pia walitaka kujifunza zaidi, ikidokeza kuwa elimu ya awali inaweza pia kukuza ujasiri na nia zaidi katika taarifa za kisayansi. Aidha, watu katika maeneo yasiyo na miji mingi walikuwa na uwezekano mkubwa wa kuripoti kuwa michoro hiyo iliwasaidia kuelewa kwa nini watafiti wanataka kusoma DNA, jambo linaloendana na hili kuwa ni mara yao ya kwanza kukutana na dhana hizi. Vilevile, tuliona tofauti za kijinsia katika ushiriki na tafsiri, ikidokeza kuwa mapendeleo ya kujifunza na mtazamo wa umuhimu wa mada unaweza kutofautiana kulingana na majukumu ya kijamii na uzoefu, jambo ambalo limetambuliwa katika machapisho yaliyopita [60,61].

Utafiti wetu una upungufu kadhaa. Kwanza, ingawa michoro ilijaribiwa kwa majaribio ya awali katika jamii zote mbili za Turkana na Orang Asli, uamuzi wetu wa kuhamia kwenye michoro mahususi kwa jamii ulisababisha tathmini rasmi kufanywa tu na washiriki wa Orang Asli, jambo ambalo lilitufanya tusiweze kulinganisha ufanisi wa michoro kati ya idadi tofauti ya watu. Pili, mahojiano yalitegemea tathmini ya washiriki wenyewe ya nyuma badala ya vipimo vya msingi vya lengo, jambo ambalo linaweza kuleta upendeleo wa kumbukumbu [62]. Hatimaye, kwa sababu matoleo ya mwisho ya michoro yaliwasilishwa kama mawasilisho ya mubashara na wasaidizi wa utafiti wa OA HeLP, mabadiliko kidogo ya maneno katika mawasilisho yote yanaweza kuwa yameathiri majibu. Hata hivyo, tunaona unyumbufu huu kama nguvu badala ya udhaifu, ukiakisi hali halisi ya ulimwengu ambapo ushiriki ni wa kimahusiano, mwingiliano, na unaoweza kubadilika, badala ya kuwa wa kiwango kimoja.

Kwa upana zaidi, vizuizi vya picha vyenyewe vina upungufu wa asili. Kwa mfano, wanajamii kadhaa walionyesha nia ya matoleo ya michoro ya uhuishaji wakidokeza kuwa mjongeo na usimulizi unaweza kuongeza zaidi uwazi kwa michakato tata ya kibayolojia.

Ingawa uhuishaji ungeruhusu maelezo yenye nguvu zaidi, kuzalisha nyenzo za uhuishaji za hali ya juu kunahitaji rasilimali nyingi za kifedha, utaalamu wa kiufundi, na miundombinu ya teknolojia inayotegemeka. Zaidi ya hayo, miundo ya ushiriki lazima iwe mifupi; kulingana na uzoefu wetu, wanajamii hawana uwezekano wa kujishughulisha na nyenzo zenye urefu wa zaidi ya dakika 10-15. Mazingatio haya yanatilia mkazo usawa ambao watafiti lazima waufikie kati ya zana bora za mawasiliano na vikwazo vya kivitendo vya muda, ufadhili, na muktadha wa ndani. Badala ya kutumika kama maelezo ya kina ya utafiti yenyewe, michoro inaeleweka vyema kama pointi za ufikiaji zinazoweza kuchochea majadiliano zaidi na mazungumzo yanayoendelea.

Licha ya upungufu huu, tunatumai kuwa kuripoti changamoto na taratibu zinazohusika katika kazi hii kunatoa mwongozo kwa wengine. Tumebainisha athari tatu za kivitendo, ambazo kwa kiasi kikubwa zinaakisi mada za awali [19, 20, 30].

1. **Nyenzo za mawasiliano madhubuti**: Zinapaswa kuchukuliwa kama rasilimali zinazokua badala ya bidhaa zilizokamilika, huku muda na rasilimali zikitengwa kwa ajili ya marekebisho ya kurudia-rudia. Tunakiri (na tuliona) kwamba hili linaweza kuwa changamoto kutokana na ugumu wa kupata rasilimali maalum (kwa mfano, ufadhili wa ruzuku) kwa ajili ya kazi hiyo.
2. **Urekebishaji mahususi wa jamii**: Unapaswa kuchukuliwa kama kipengele muhimu katika muundo wa mchoro.
3. **Tathmini ya nyenzo za ushiriki**: Ni muhimu kutathmini nyenzo si tu kwa uelewaji, bali pia ikiwa zinaendana na maslahi ya washiriki, maadili, na malengo ya kushirikiana na watafiti.

Tayari tumeanza kutumia kanuni hizi katika mipango mingine ya OA HeLP na THGP, ikiwa ni pamoja na juhudi za hivi karibuni za kurejesha matokeo. Wakati utafiti wa kigenomu ukiendelea kufanyika kando ya idadi ya watu asilia ambao kihistoria hawajawakilishwa vya kutosha, mbinu kama hizi zinalenga kutoa njia ya kujenga uaminifu na kukuza uelewaji wa pande zote mbili.

**Taarifa ya upatikanaji wa data**

Kipaumbele cha juu cha OA HeLP ni kupunguza hatari kwa washiriki wa utafiti. OA HeLP inafuata 'Kanuni za CARE za Utawala wa Data ya Asili' (Manufaa ya Pamoja, Mamlaka ya Kudhibiti, Uwajibikaji, na Maadili). OA HeLP pia imejitolea kufuata 'Kanuni Mwongozo za FAIR za usimamizi na uongozi wa data za kisayansi' (Inayopatikana, Inayofikika, Inayoweza kushirikiana, Inayoweza kutumika tena). Ili kuzingatia kanuni hizi huku tukipunguza hatari, data ya kiwango cha mtu binafsi imehifadhiwa katika hazina ya data iliyolindwa ya OA HeLP, na inapatikana kupitia ufikiaji wenye vikwazo.

Maombi ya data ya kiwango cha mtu binafsi yasiyo na utambulisho yanapaswa kuwa katika muundo wa maombi yanayoezea kwa kina matumizi sahihi ya data na maswali ya utafiti yatakayoshughulikiwa, taratibu zitakazotumika kwa ajili ya usalama wa data na faragha ya mtu binafsi, manufaa yanayoweza kupatikana kwa jamii zinazofanyiwa utafiti, na taratibu za kutathmini na kupunguza tafsiri zinazoweza kuleta unyanyapaa wa matokeo ya utafiti. Maombi ya data ya kiwango cha mtu binafsi yasiyo na utambulisho yatahitaji idhini ya taasisi ya IRB (hata kama imesamehewa). OA HeLP imejitolea kwa sayansi huria na uongozi wa mradi unapatikana kusaidia wachunguzi wanaovutiwa katika kuandaa maombi ya ufikiaji wa data (tazama orangaslihealth.org kwa maelezo zaidi na taarifa za mawasiliano).

Muhtasari wa data yote iliyowasilishwa upo katika Nyenzo za Nyongeza. Misimbo zilizotumiwa kwa uchambuzi huu zinaweza kupatikana kwenye GitHub (<https://github.com/audreyarner/genetic_illustrations>).

**Shukrani**

Zaidi ya yote, tunawashukuru washiriki wa Orang Asli na Turkana ambao wameturuhusu kwa ukarimu kufanya kazi katika jamii zao, pamoja na ukarimu wao na msaada kwa mradi huu. Pia tunawashukuru wanachama wa Mradi wa Afya na Mitindo ya Maisha ya Orang Asli (OA HeLP) na Mradi wa Afya na Jenetiki wa Turkana (THGP) waliopitia matoleo ya awali ya michoro hiyo. Pia tunashukuru kwa Jada Benn Torres na wanachama wa Lea Lab kwa maoni na msaada wao.

**Ufadhili**

AMA alisaidiwa na Mpango wa Udhamini wa Utafiti wa Wahitimu wa Shirika la Kitaifa la Sayansi (1937963 & 2444112) na Ruzuku ya Kuboresha Tasnifu ya Udaktari (2419584), pamoja na Ruzuku ya Kazi ya Nyanjani ya Tasnifu ya Wenner-Gren, Ruzuku ya Utafiti ya Leakey Foundation, na Tuzo ya Vanderbilt ya Ugunduzi wa Udaktari. Pia tunashukuru Mpango wa Masomo ya Mageuzi wa Vanderbilt kwa msaada wao wa kifedha. AJL, IW, na TSK walisaidiwa na Shirika la Kitaifa la Sayansi (Anthropolojia ya Kibayolojia 2142090).
