# Supplementary material for: Development and Assessment of Tailored Illustrations to Enhance Community Understandings of Genetics Topics
Source: Am J Biol Anthropol. 2026 Jul 20;190(3):e70314. doi: 10.1002/ajpa.70314 (PMC13385646; doi:10.1002/ajpa.70314)
Supplement: Supplementary file 5 — Supporting Information: S1. English interview. Supporting Information: S2. English pamphlet. Supporting Information: S3. Malay pamphlet. Supporting Information: S4. Swahili translation of full text. Figure S1: Thematic map for the information participants thought was in their blood prior to seeing the illustrations. Figure S2: Thematic map for responses about what each participant learned. Figure S3: Thematic map for responses about why a chosen image was the participant's favorite. Figure S4: MCA scree plot. Table S1: Demographic information for participants taking final interview. Table S2: Percent interrater agreement for codes. Table S3: Description of each illustrated concept. Table S4: General topics discussed for each image. Table S5: Genetics‐related topics participants expressed further interest in. Table S6: Binomial model results for question response ~1. Table S7: MCA dimension model results as a function of sex, age, highest education level, and urbanicity score. Table S8: Question response model results as a function of sex, age, highest education level, and urbanicity score. Table S9: Question response model results as a function of sex, age, binary of any formal education, and urbanicity score. Table S10: Question response model results as a function of ethnolinguistic group, sex, age, binary of any formal education, and urbanicity score. [file AJPA-190-e70314-s005.docx]

Supplemental materials for **“Development and assessment of tailored illustrations to enhance community understandings of genetics topics”**

This supplement consists of:

**Supplementary Methods**

**Supplementary Information**

- Supplementary Information 1. English Interview
- Supplementary Information 2. English Pamphlet
- Supplementary Information 3. Malay Pamphlet
- Supplementary Information 4. Swahili translation of full text

**Supplementary Figures**

- Supplementary Figure 1. Thematic map for the information participants thought was in their blood prior to seeing the illustrations
- Supplementary Figure 2. Thematic map for responses about what each participant learned.
- Supplementary Figure 3. Thematic map for responses about why a chosen image was the participant’s favorite.
- Supplementary Figure 4. MCA scree plot

**Supplementary Tables**

- Supplementary Table 1. Demographic information for participants taking final interview
- Supplementary Table 2. Percent interrater agreement for codes
- Supplementary Table 3. Description of each illustrated concept
- Supplementary Table 4: General topics discussed for each image
- Supplementary Table 5. Genetics-related topics participants expressed further interest in
- Supplementary Table 6. Binomial model results for question response ~ 1
- Supplementary Table 7. MCA dimension model results as a function of sex, age, highest education level, and urbanicity score
- Supplementary Table 8. Question response model results as a function of sex, age, highest education level, and urbanicity score
- Supplementary Table 9: Question response model results as a function of sex, age, binary of any formal education, and urbanicity score
- Supplementary Table 10: Question response model results as a function of ethnolinguistic group, sex, age, binary of any formal education, and urbanicity score

Supplementary Methods

*Urbanicity score generation*

Orang Asli live across a wide lifestyle gradient, which we have previously described using a location-based “urbanicity” score [[1,2]](https://paperpile.com/c/XHij6a/9pNk+fNXQ). Using a location-level scale is advantageous because it gives a representation of the resources available across the community (e.g., an individual may not own a television but may have access through families or friends). To quantify individuals’ exposure to urban centers and urban infrastructure, we used a scale first proposed by Novak et al [[3]](https://paperpile.com/c/XHij6a/qS3hV). This scale was tested in Orang Asli and predicted cardiometabolic health better than other measures of urbanicity. Scale construction is shown below and population density was estimated from NASA’s Gridded Population of the World resource with a resolution of 2.5 arc-minutes [[4]](https://paperpile.com/c/XHij6a/gFE4R). The urbanicity scores values ranged from 13.802-24.636, modeled as a continuous variable in linear models during our analysis.

| **Population density (estimated number of people per square kilometer)** | **Contribution to scale** |
| --- | --- |
| 0-100 | 1 |
| 100-200 | 2 |
| 200-300 | 3 |
| 300-400 | 4 |
| 400-500 | 5 |
| 500-1000 | 6 |
| 1000-2000 | 7 |
| 2000-3000 | 8 |
| 3000-4000 | 9 |
| 4000-6000 | 10 |
| 6000-8000 | 11 |
| 8000-10000 | 12 |
| 10000-15000 | 13 |
| 15000-20000 | 14 |
| >20000 | 15 |
| **Occupation** |  |
| Proportion of the population involved in non-wage labor | 10 - (10 x (proportion)) |
| **Built Environment** |  |
| Proportion of households with flush toilets | 5 x proportion |
| Proportion of households with electricity | 5 x proportion |
| **Communication/market-derived items** |  |
| Proportion of households with mobile phone | 5 x proportion |
| Proportion of households with television | 5 x proportion |
| **Education** |  |
| Proportion of surveyed individuals > 40 years with any level of formal education | 10 x proportion |
| Proportion of surveyed individuals < 40 years with any level of formal education | 10 x proportion |


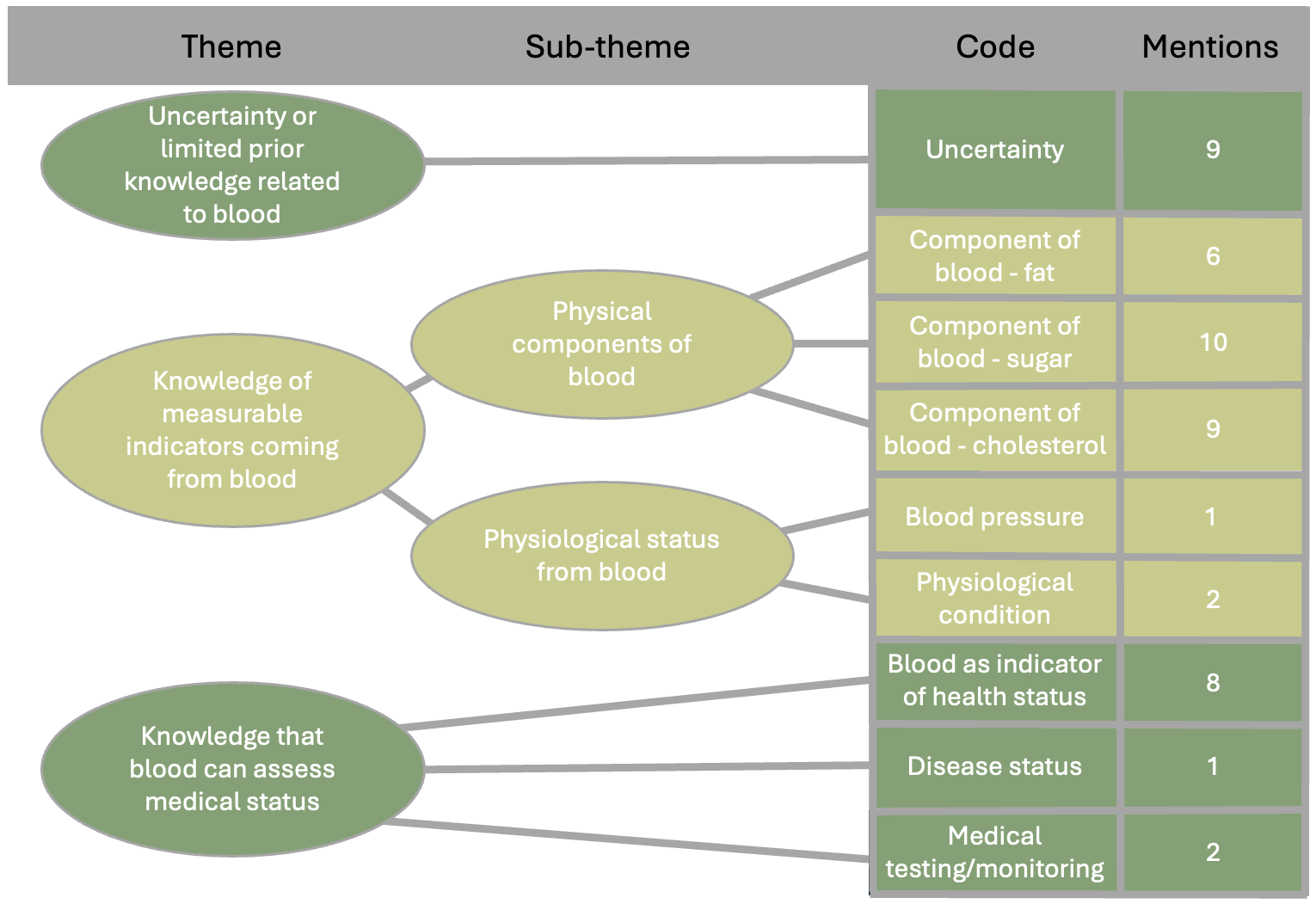


**SI Figure 1. Thematic map for the information participants thought was in their blood prior to seeing the illustrations.** Each theme and sub-theme are listed, as well as the codes associated with each. Mentions refers to the total number of times a code was recorded across all individuals.


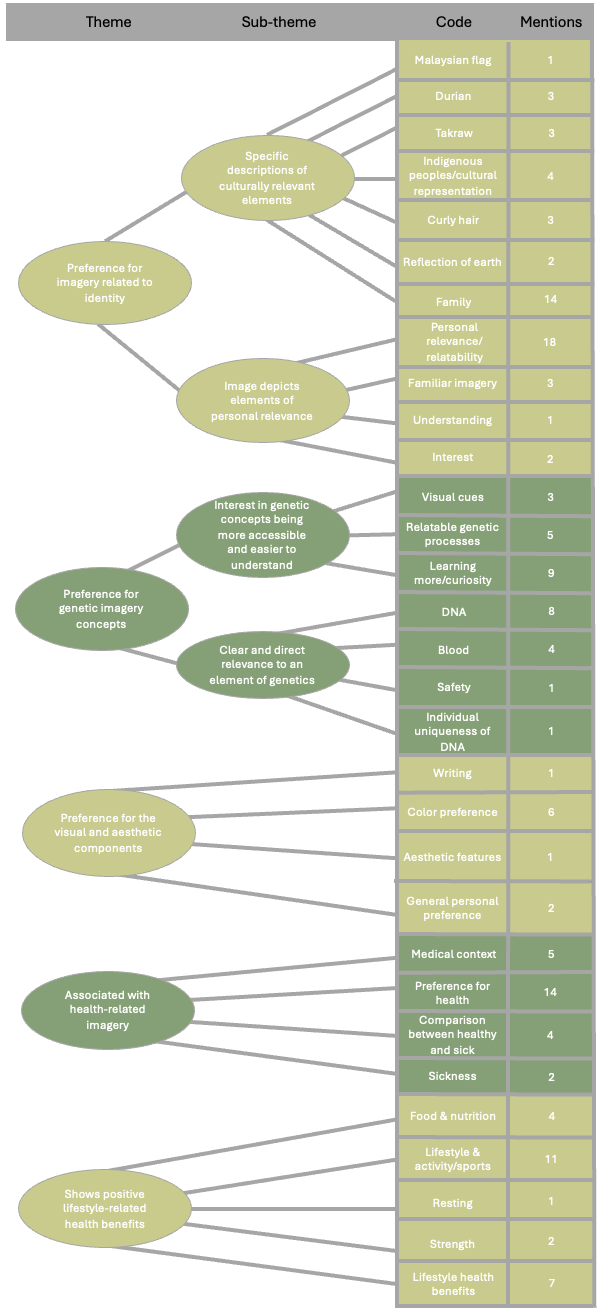


**SI Figure 2. Thematic map for responses about what each participant learned.** Each theme and sub-theme are listed, as well as the codes associated with each. Mentions refers to the total number of times a code was recorded across all individuals.


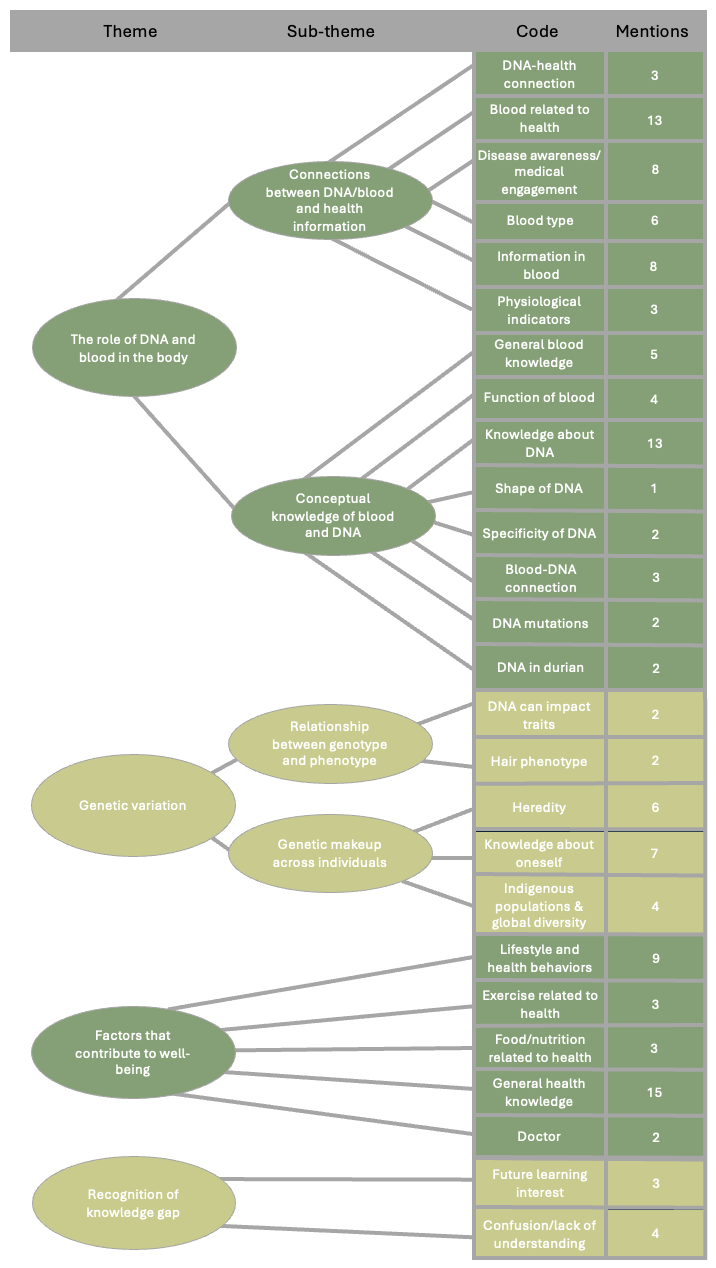


**SI Figure 3. Thematic map for responses about why a chosen image was the participant’s favorite.** Each theme and sub-theme are listed, as well as the codes associated with each. Mentions refers to the total number of times a code was recorded across all individuals.

**
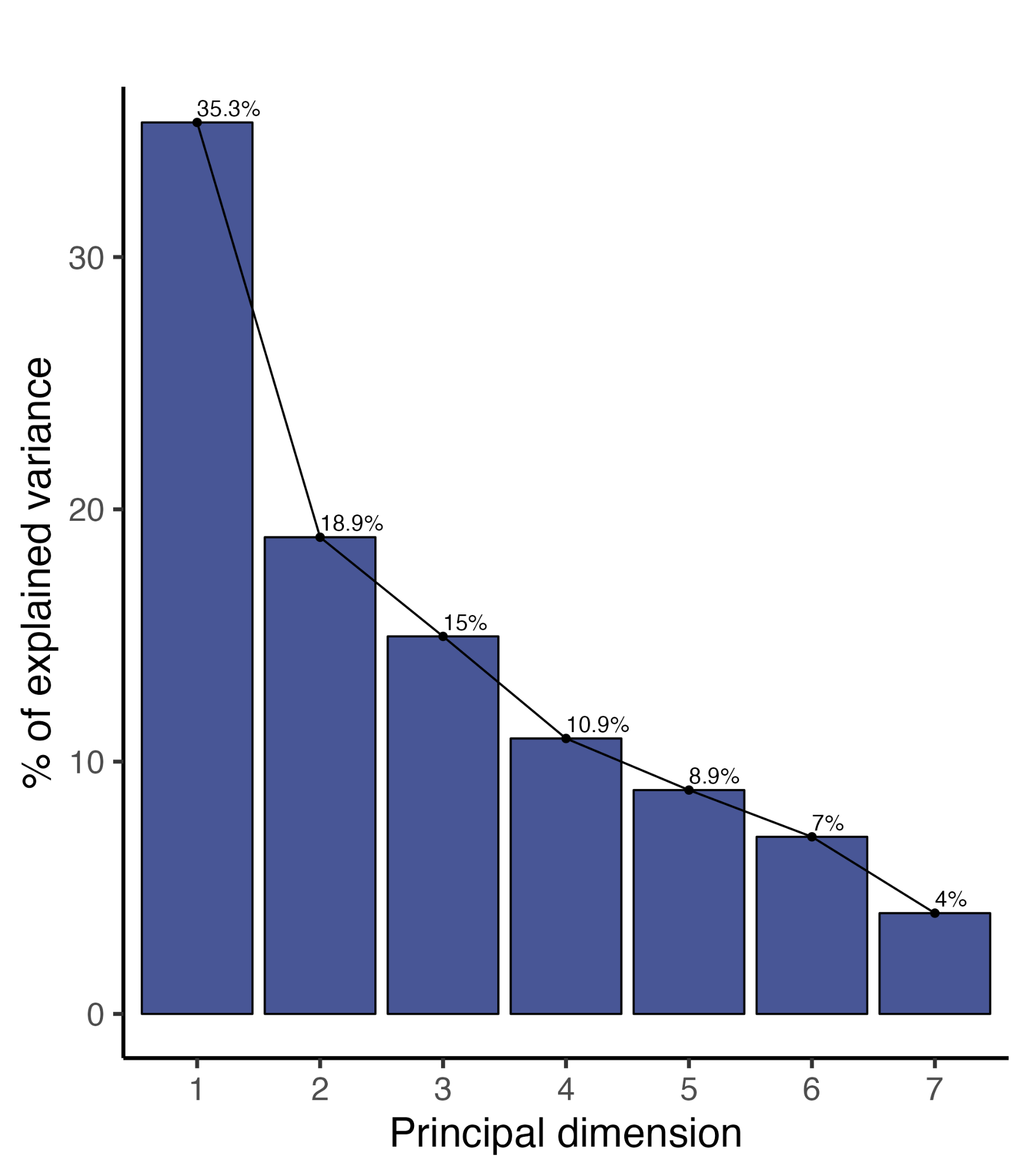
**

**SI Figure 4. MCA scree plot.** Scree plot showing the proportion of variance explained by the top six dimensions of the multiple correspondence analysis (MCA).

SI Table 1: Demographic information for participants taking final interview

| **Location** | **#**  **participants** | **Ethnolinguistic group** | **Sex** | | **Urbanicity score** | **Highest level of formal education** | | | |
| --- | --- | --- | --- | --- | --- | --- | --- | --- | --- |
|  |  |  | **# F** | **# M** |  | **None** | **Primary school** | **Secondary school** | **University** |
| A | 21 | Temiar | 8 | 13 | 19.156 | 0 | 11 | 9 | 1 |
| B | 10 | Semai | 8 | 2 | 24.691 | 0 | 3 | 7 | 0 |
| C | 17 | Semai | 13 | 4 | 24.636 | 12 | 3 | 2 | 0 |
| D | 20 | Semai | 11 | 9 | 13.802 | 12 | 3 | 5 | 0 |
| E | 8 | Temiar | 8 | 0 | 21.920 | 1 | 2 | 5 | 0 |
| F | 16 | Temiar | 5 | 11 | 19.645 | 6 | 5 | 5 | 0 |

SI Table 2: Percent interrater agreement for codes

| **Question** | **Percent interrater agreement** |
| --- | --- |
| What type of information did you think was in your blood prior to seeing the images? | 100% |
| Why was that image your favorite? | 85.7% |
| What is one thing you learned after seeing the illustrations? | 86.2% |

SI Table 3: Description of each illustrated concept

| **Question** | **Concept illustrated** | **Example of Orang Asli imagery used** |
| --- | --- | --- |
| What is DNA? | DNA carries information inherited from parents and shared across living things | Hair texture variation among family members, durian varieties |
| Can DNA affect your health? | Some DNA changes have effects on health while most do not | Traditional clothing, housing, and sleep mat |
| What can scientists learn from DNA? | How changes in DNA relate to health and ancestry | Picture of Orang Asli along with other Indigenous populations |
| Besides DNA, what else do you study in my blood? | Blood contains measurable components like sugars and fats | Nasi lemak (traditional dish) with less sugars than a burger in the city |
| Why are scientists interested in markers of health? | Links between lifestyle change and biomarkers | Physical activity of an individual playing takraw (traditional ball game) |
| Who has access to my DNA? | Data access and research ethics | Include Kuala Lumpur (capital city of Malaysia) in the background |

SI Table 4: General topics discussed for each image

| **Image** | **Question** | **Concepts discussed** |
| --- | --- | --- |
| A | What is DNA? | - To learn about health, we draw blood - Within blood is a special material called DNA - DNA is in every part of your body, and has instructions that influence how your body looks and works - Scientists can study the DNA in a person’s blood to learn about your entire body |
| B | What is DNA? | - Every person’s DNA is different, which is why we do not all look the same - Example of hair texture: DNA carries information that impacts the texture of your hair - We get our DNA from our mother and father, and they get their DNA from their parents - The passing down of DNA is called “inheritance” and is why you have similar traits to your relatives - Even though you inherit your DNA, your DNA is unique and different from every other person’s |
| C | What is DNA? | - All plants and animals also have DNA - Example: Differences in DNA contribute to how each durian variety has a unique look, smell, and taste - The environment also impacts how durian looks, smells, and tastes - DNA and the environment interact to impact traits |
| D | Can DNA affect your health? | - DNA can affect your health - When DNA is inherited, there can be small changes called “mutations” - Most mutations do not have a major influence on how you look or how your body works - Sometimes mutations can cause disease |
| E | What can scientists learn from DNA? | - The DNA of other populations, including Indigenous populations, has been studied to understand how humans adapt to their environments - DNA can also be used to tell how different populations around the world are related to one another (e.g., which populations have a shared history) - We can also understand the mutations that cause health problems - Most research has been done in the United States and Europe, but these studies do not consider the enormous diversity of DNA that exists among people living in other parts of the world |
| F | Besides DNA, what else do you study in my blood? | - There are biomarkers in your blood, such as sugars, fats, and cholesterol, that we measure to learn about health - Some of these biomarkers can come from the food you eat - Too much sugar or fat can result in negative health outcomes like diabetes and cardiovascular disease - Oftentimes the traditional foods eaten by Orang Asli have less saturated fats and sugar than food in cities - When we take your blood we can measure your blood sugar and fat levels right away and tell you if you have healthy levels or not |
| G | Why are scientists interested in markers of health? | - Some scientists are interested in how lifestyle impacts health, measured through blood biomarkers - Traditionally, many people have lived off the land and forest around them, but now people around the world have moved to environments where they are less active and are sedentary - There are large lifestyle differences between Orang Asli living traditionally in the rainforest versus Orang Asli who are living in cities |

SI Table 5: Genetics-related topics participants expressed further interest in

| **Topic** | **Individuals** |
| --- | --- |
| Health and disease | 45 |
| Relatedness | 42 |
| Specifics about DNA | 17 |
| Similarities and differences to other populations | 26 |
| Other | 13 |

SI Table 6: Binomial model results for question response ~ 1

| **Question** | **P-value** | **FDR** |
| --- | --- | --- |
| 1. I think I know more about DNA now | 7.48x10^-8^ | 1.2x10^-7^ |
| 2. The illustrations helped me understand more about genetics | 6.82x10^-10^ | 2.73x10^-9^ |
| 3. The illustrations helped me understand why researchers want to study DNA | 1.47x10^-8^ | 2.94x10^-8^ |
| 4. I could explain the illustrations to a friend | 5.45x10^-4^ | 6.23x10^-4^ |
| 5. I would look at the illustrations again | 1.18x10^-9^ | 3.15x10^-9^ |
| 6. I would tell a friend to look at the illustrations | 1.04x10^-7^ | 1.38x10^-7^ |
| 7. I would like to learn more about genetics | 5.18x10^-10^ | 2.73x10^-9^ |
| 8. The illustrations were hard to understand | 4.08x10^-3^ | 4.08x10^-3^ |

SI Table 7: MCA dimension model results as a function of sex, age, highest education level, and urbanicity score

| **Outcome** | **Covariate** | **Beta** | **SE** | **P-value** | **FDR** |
| --- | --- | --- | --- | --- | --- |
| Dimension 1 | sex | -0.1299 | 0.1291 | 0.3172 | 0.3966 |
| Dimension 1 | age | -0.0003 | 0.0058 | 0.9575 | 0.9575 |
| Dimension 1 | highest education level | -0.1654 | 0.0897 | 0.0686 | 0.1267 |
| Dimension 1 | urbanicity score | -0.0287 | 0.0159 | 0.0760 | 0.1267 |
| Dimension 2 | sex | -0.1279 | 0.0954 | 0.1835 | 0.4083 |
| Dimension 2 | age | -0.0005 | 0.0043 | 0.9024 | 0.9024 |
| Dimension 2 | highest education level | -0.0407 | 0.0662 | 0.5402 | 0.6753 |
| Dimension 2 | urbanicity score | 0.0243 | 0.0118 | 0.0422 | 0.2113 |

SI Table 8: Question response model results as a function of sex, age, highest education level, and urbanicity score


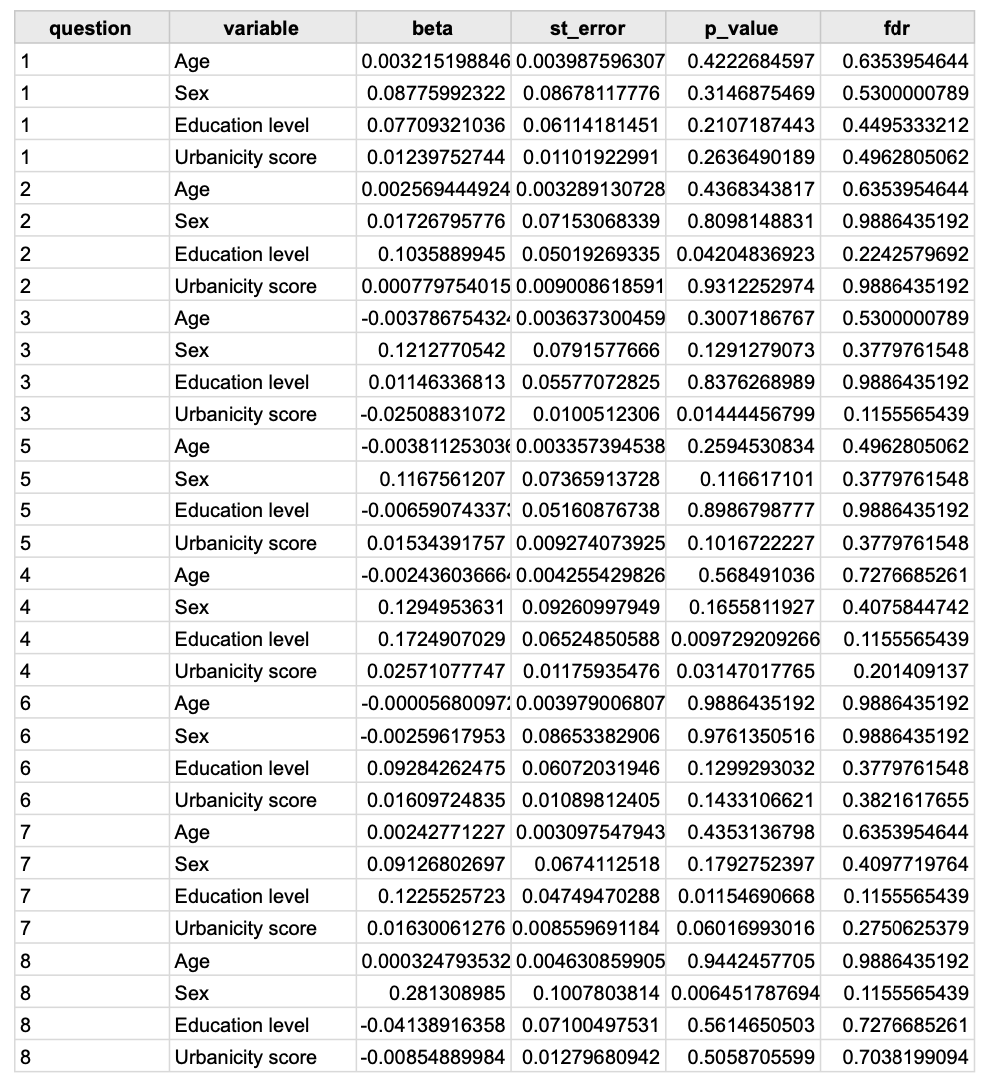


SI Table 9: Question response model results as a function of sex, age, binary of any formal education, and urbanicity score


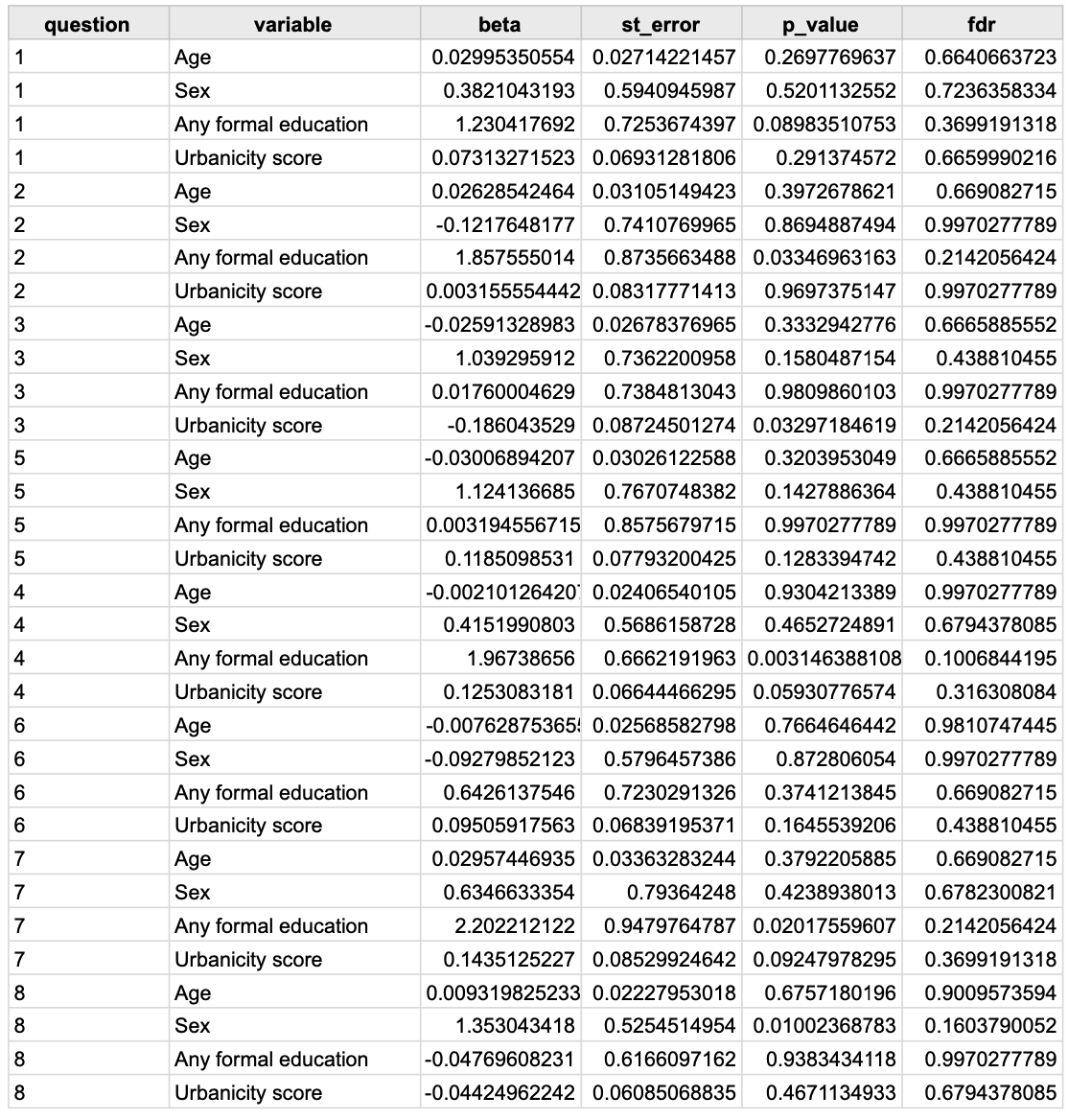


SI Table 10: Question response model results as a function of ethnolinguistic group, sex, age, binary of any formal education, and urbanicity score


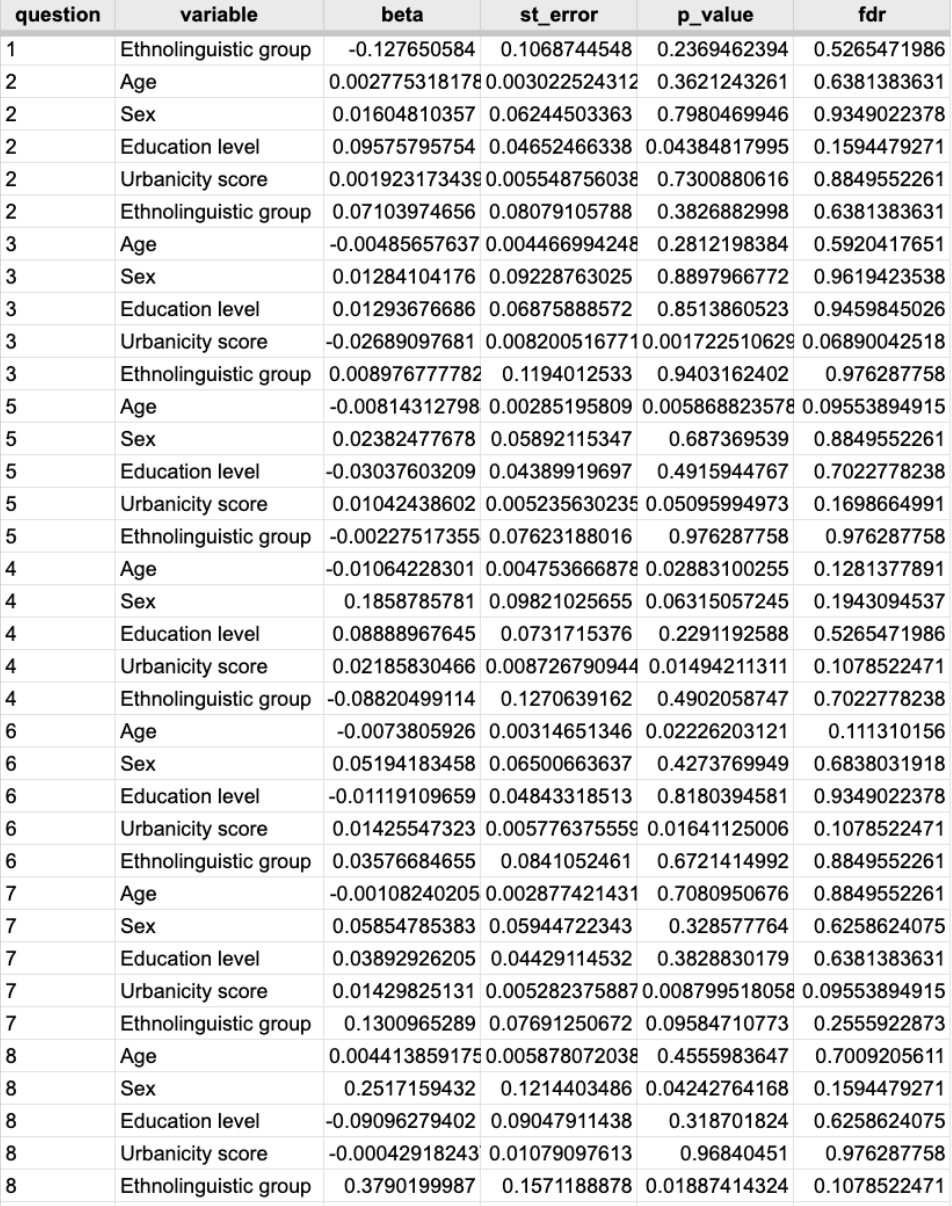


Works Cited

1. [Watowich MM, Arner AM, Wang S, John E, Kahumbu JC, Kinyua P, et al. The built environment is more predictive of cardiometabolic health than other aspects of lifestyle in two rapidly transitioning Indigenous populations. medRxiv. 2024. doi:](http://paperpile.com/b/XHij6a/9pNk)[10.1101/2024.08.26.24312234](http://dx.doi.org/10.1101/2024.08.26.24312234)

2. [Kraft TS, Wallace IJ, Lim YAL, Tam KL, Huat TBTATB, Tennyson R, et al. Physical activity and cardiometabolic health across an extreme lifestyle gradient. Public and Global Health. medRxiv; 2025. Available:](http://paperpile.com/b/XHij6a/fNXQ) <https://www.medrxiv.org/content/10.1101/2025.07.11.25331394v1>

3. [Novak NL, Allender S, Scarborough P, West D. The development and validation of an urbanicity scale in a multi-country study. BMC Public Health. 2012;12: 530.](http://paperpile.com/b/XHij6a/qS3hV)

4. [Center For International Earth Science Information Network-CIESIN-Columbia University. Gridded population of the world, version 4 (GPWv4): Population count, revision 11. Palisades, NY: NASA Socioeconomic Data and Applications Center (SEDAC); 2018. doi:](http://paperpile.com/b/XHij6a/gFE4R)[10.7927/H4JW8BX5](http://dx.doi.org/10.7927/H4JW8BX5)
